# Single-cell and machine learning-based pyroptosis-related gene signature predicts prognosis and immunotherapy response in glioblastoma

**DOI:** 10.3389/fimmu.2025.1693940

**Published:** 2025-10-24

**Authors:** Liren Fang, Desheng Wang, Fanlei Meng, Yinzhi Wang, Lu Feng, Hong Li

**Affiliations:** ^1^ Neurosurgery Department, Second Hospital of Tianjin Medical University, Tianjin, China; ^2^ Neurosurgery Department, Tianjin Hospital, Tianjin, China; ^3^ Neurosurgery Department, Taizhou Central Hospital (Taizhou University Hospital), Zhejiang, China

**Keywords:** glioblastoma, pyroptosis, machine learning, prognostic signature, SPP1 signaling

## Abstract

**Background:**

Glioblastoma (GBM) is the most aggressive primary malignancy of the central nervous system, characterized by profound heterogeneity and an immunosuppressive microenvironment, leading to dismal prognosis. Pyroptosis, an inflammatory form of programmed cell death, has been increasingly linked to tumor immunity and progression; however, its molecular roles and clinical implications in GBM remain insufficiently understood.

**Methods:**

We integrated bulk transcriptome profiles from TCGA-GBM, CGGA, and GEO datasets with single-cell RNA sequencing data from GSE141383 and GSE223063. A comprehensive GBM single-cell atlas was constructed using Seurat and Harmony, and malignant epithelial cells were inferred via inferCNV. Pyroptosis activity was quantified by five complementary algorithms, while Monocle2 and Slingshot were employed for pseudotime trajectory reconstruction, and SCENIC was applied for transcription factor network analysis. Candidate prognostic genes identified from malignant epithelial subsets were further used to develop a Pyroptosis-Related Gene Signature (PRGS) through a systematic evaluation of ten machine learning algorithms and their combinations, with subsequent validation across multiple cohorts. Functional enrichment (GSVA, GSEA), tumor microenvironment estimation (ESTIMATE, ssGSEA), drug sensitivity prediction (GDSC2), and *in vitro* experiments were performed to characterize the biological and therapeutic relevance of PRGS, with *MAP1B* selected for experimental validation.

**Results:**

Single-cell analyses revealed heterogeneous pyroptosis activity across GBM cell populations. Distinct ligand–receptor communications were observed between high- and low-pyroptosis groups, among which the SPP1-centered signaling axis showed pronounced remodeling, suggesting a pivotal role in tumor–immune crosstalk. Pseudotime and regulatory network analyses of malignant epithelial cells further delineated differentiation trajectories and transcriptional regulators. The PRGS, established by StepCox[both]+Ridge modeling, demonstrated robust prognostic stratification and predictive power across independent datasets. High PRGS scores were consistently associated with poorer survival outcomes, higher TIDE scores, and reduced IPS values, indicating enhanced immune evasion and attenuated immunotherapy benefit. Enrichment analyses highlighted that high PRGS tumors were linked to metabolic reprogramming and DNA repair pathways, whereas low PRGS tumors exhibited signatures of immune activation. Drug sensitivity analyses revealed distinct therapeutic vulnerabilities between subgroups. Functional assays confirmed that *MAP1B* promotes proliferation, migration, and invasion in GBM cells, reinforcing its oncogenic role.

**Conclusion:**

This study systematically elucidates the role of pyroptosis in GBM and establishes PRGS as a reliable prognostic biomarker. PRGS not only refines risk stratification and predicts immunotherapy response but also provides molecular insights into tumor metabolism and immune regulation, thereby offering potential avenues for targeted therapeutic strategies in GBM.

## 1 Introduction

GBM is the most common and highly aggressive primary brain tumor in adults, accounting for more than 50% of all gliomas (1). It is characterized by marked invasiveness and profound intra- and inter-tumoral heterogeneity, leading to rapid progression, therapeutic resistance, and frequent recurrence (2, 3). Despite the current standard of care consisting of maximal surgical resection followed by radiotherapy and temozolomide chemotherapy, the median overall survival of GBM patients remains less than 15 months, with a five-year survival rate below 7% (4, 5). Although immunotherapies such as immune checkpoint inhibitors have shown remarkable efficacy in several solid tumors, their benefits in GBM have been limited, largely due to the profoundly immunosuppressive tumor microenvironment (6, 7). These challenges highlight the urgent need to identify novel molecular mechanisms and biomarkers that can improve risk stratification, therapeutic prediction, and the development of effective treatment strategies.

Pyroptosis, a form of programmed cell death mediated by inflammasome activation and gasdermin pore formation, is distinguished by cell membrane rupture and the release of proinflammatory cytokines such as IL-1β and IL-18 (8–11). Unlike apoptosis, pyroptosis elicits a robust inflammatory response that can reshape the tumor microenvironment (12, 13). Previous studies have indicated its dual role in cancer biology: on the one hand, pyroptosis can suppress tumor progression by enhancing immune infiltration and antitumor immunity; on the other hand, excessive or dysregulated pyroptosis may drive chronic inflammation, immune evasion, and malignant progression (14–16). While pyroptosis has been investigated in breast, colorectal, and hepatocellular carcinomas, its activity patterns, regulatory mechanisms, and prognostic significance in GBM remain poorly understood.

The advent of single-cell RNA sequencing (scRNA-seq) has greatly advanced our understanding of tumor complexity (17, 18). Unlike bulk RNA-seq, which provides averaged gene expression across populations, scRNA-seq enables the dissection of cellular heterogeneity at single-cell resolution, allowing the identification of key subpopulations, intercellular communication networks, and developmental trajectories (19). Recent studies have applied scRNA-seq to GBM and revealed distinct immune cell subsets as well as glial cell states associated with therapeutic resistance and invasion, underscoring its value in uncovering novel biological mechanisms and potential therapeutic targets (20, 21). At the same time, integrating large-scale transcriptomic data with machine learning–based modeling has emerged as a powerful strategy in cancer research, offering robust tools for biomarker discovery and prognostic assessment across multiple cohorts (22).

In this study, we integrated scRNA-seq and bulk RNA-seq datasets of GBM to comprehensively evaluate pyroptosis activity and its influence on malignant epithelial cell differentiation and signaling pathways. Building upon these findings, we developed and validated a PRGS using a systematic machine learning framework and assessed its prognostic value and predictive potential for immunotherapy response and drug sensitivity. Furthermore, we experimentally validated the role of a key gene, *MAP1B*, in GBM cell lines. Collectively, our work provides novel insights into the biological functions of pyroptosis in GBM and establishes PRGS as a promising tool for risk stratification and precision therapy.

## 2 Methods

### 2.1 Collection and integration of data

Transcriptomic profiles and clinical annotations were obtained from publicly accessible repositories. Bulk RNA-seq data included the glioblastoma cohort from The Cancer Genome Atlas (TCGA-GBM, expression values transformed into TPM format) and the Chinese Glioma Genome Atlas (CGGA, http://www.cgga.org.cn/). Additional validation cohorts were retrieved from the Gene Expression Omnibus (GEO, https://www.ncbi.nlm.nih.gov/geo/), including GSE13041, GSE74187, and GSE83300. To minimize technical heterogeneity among datasets, batch effects were corrected using the sva package in R (23). scRNA-seq data were also collected from GEO. The GSE141383 dataset comprises nine human glioma surgical specimens, whereas GSE223063 includes six glioblastoma samples from three patients. For immunotherapy response evaluation, the immunophenoscore (IPS) was obtained from The Cancer Immunome Atlas (TCIA, https://tcia.at/home) (24).Furthermore, publicly available immunotherapy-related gene sets curated from prior studies were integrated for downstream analyses. Detailed dataset-level metadata (source/accession, pathological type, and sample size) are summarized in **Supplementary Table S1**.

All datasets were publicly available, and thus no additional ethical approval was required.

### 2.2 Identification and functional profiling of single-cell populations

After importing raw scRNA-seq profiles from multiple samples, the data were merged into a combined matrix. During quality assessment, cells suspected to be doublets as well as those exhibiting disproportionately high mitochondrial content were removed to ensure reliability.

The Seurat workflow was applied for downstream processing (25, 26). Prior to normalization, quality control (QC) filters were applied: cells with a mitochondrial gene percentage > 40% were removed; genes detected in fewer than three cells were excluded; and outlier cells by nFeature_RNA/nCount_RNA were filtered using a robust median absolute deviation (MAD) criterion. Putative doublets were identified and removed with DoubletFinder (integrated with the Seurat workflow), with the expected doublet rate estimated from capture loading and the classification parameter (pK) selected via a standard parameter-sweep procedure. Following normalization and identification of variable features, dimensionality reduction was performed with principal component analysis. To mitigate inter-sample variation, Harmony (27) was employed for batch correction. Clustering and visualization were subsequently conducted using UMAP/t-SNE. Marker genes specific to each cluster were determined, and cell identities were annotated by integrating canonical references.

Functional characteristics of each cluster were further investigated using the SCP package, which enabled enrichment analyses to delineate potential biological roles of distinct subpopulations.

### 2.3 Pyroptosis scoring and stratification

To assess the activity of pyroptosis at the single-cell level, a curated gene set was compiled based on previous studies (25). Multiple computational strategies were applied to the integrated scRNA-seq object, including AUCell, UCell, singscore, ssGSEA, and AddModuleScore. AUCell estimated enrichment through area under the curve (AUC) values derived from ranked gene expression; UCell and singscore generated rank-based scores for each cell; ssGSEA was implemented via the GSVA framework to compute pathway enrichment; and AddModuleScore from Seurat provided additional module-level scoring. Scores obtained from these five approaches were standardized (z-score normalization and min–max scaling) and combined into a composite index termed Scoring, representing the overall pyroptosis activity of each cell. This score was appended to the Seurat metadata for subsequent visualization and downstream analyses.

Distribution of pyroptosis scores was illustrated using UMAP and dot plots, while violin plots highlighted inter-cluster differences. Cells were stratified into Pyroptosis_high and Pyroptosis_low groups according to the median Scoring value, which served as the basis for subsequent cell–cell communication analysis.

### 2.4 Ligand–receptor network analysis

Cell–cell communication was investigated using the CellChat framework on the scRNA-seq dataset (28). Annotated cell identities were incorporated into the workflow, and a curated human ligand–receptor database was used as the reference. Overexpressed ligands and receptors were identified within each cell group and projected onto a protein–protein interaction network to infer possible intercellular communication. For each ligand–receptor pair, CellChat estimated communication probabilities and applied permutation testing to assess statistical significance. These results were subsequently aggregated to construct pathway-level signaling networks, with network centrality measures used to evaluate the relative contributions of distinct cell populations. Communication networks were established separately for Pyroptosis_high and Pyroptosis_low groups, followed by integrative comparison to detect differences in both the number and the strength of signaling interactions.

### 2.5 Determination of malignant epithelial cells

To identify malignant epithelial cells, copy number variations (CNVs) were inferred from scRNA-seq profiles using inferCNV (29). Endothelial cells were designated as the reference group, against which tumor cells were compared. Genes were ordered by chromosomal position, and hierarchical clustering was applied to visualize CNV patterns through heatmaps. Based on the inferred CNV matrix, k-means clustering was employed to group cells exhibiting pronounced copy number alterations. A CNV score was then calculated for each cell to quantify the degree of genomic abnormality. This score, in combination with clustering outcomes, enabled the classification of malignant versus non-malignant subpopulations. Cells identified as malignant were subsequently extracted and saved for downstream analyses, such as subclustering and exploration of transcriptional heterogeneity.

### 2.6 Dynamics and transcriptional regulation of malignant epithelial cells

To delineate the developmental dynamics of malignant epithelial cells, two complementary pseudotime approaches were applied. Monocle2 (30) was first used to reconstruct cell state transition trees, inferring temporal progression based on differentially expressed genes and dimensionality reduction. In parallel, Slingshot (31) was employed to fit smooth trajectories across clusters, enabling the identification of branching events and differentiation directions. The integration of these two algorithms provided consistent and robust insights into the evolutionary patterns of malignant epithelial populations. For transcriptional regulation, the SCENIC (32) workflow was implemented to reconstruct gene regulatory networks. This framework integrates co-expression analysis with cis-regulatory motif information to define regulons and assess their activity at single-cell resolution. To reduce computational complexity while maintaining representativeness, 100 cells were randomly sampled from each malignant epithelial cluster for SCENIC analysis. The results highlighted transcription factors with pivotal roles in cell fate determination and functional heterogeneity.

### 2.7 Multi-algorithm modeling and performance validation

Differentially expressed genes were first identified between pyroptosis-high and pyroptosis-low malignant epithelial cells, and those significantly associated with GBM patient survival were selected through univariate Cox regression as candidate variables.

Model development was conducted within a machine learning framework that incorporated ten algorithms: stepwise Cox regression, Lasso, Ridge, partial least squares regression for Cox (plsRcox), CoxBoost, random survival forest (RSF), generalized boosted regression modeling (GBM), elastic net (Enet), supervised principal components (SuperPC), and survival support vector machine (survival-SVM). Algorithmic combinations were systematically tested under 10-fold cross-validation, and models containing fewer than five genes after selection were excluded from comparison. Algorithmic combinations were evaluated under cross-validation; per a pre-specified rule, models with fewer than five genes were not retained.

For each cohort, risk scores were computed based on the final model, and patients were dichotomized into high- and low-risk groups according to the median value. Kaplan–Meier analysis was applied to assess survival differences between groups. Model performance was further evaluated using the receiver operating characteristic (ROC) curve and concordance index (C-index). In addition, principal component analysis (PCA) was performed to visualize patient distribution patterns, providing an intuitive validation of the model’s discriminative ability between risk categories.

### 2.8 Functional annotation via GSVA and GSEA

To characterize the biological differences between risk groups, two complementary enrichment approaches were employed. Gene set variation analysis (GSVA) was first applied to calculate enrichment scores for hallmark pathways across all GBM samples (33). Differential pathway activity between high- and low-risk groups was then assessed using the limma framework.

In parallel, gene set enrichment analysis (GSEA) was performed to capture global expression-level trends (34). Genes were ranked by log2 fold change between groups, and enrichment was tested against curated KEGG and GO gene sets. Normalized enrichment scores (NES) were computed to determine functional programs preferentially activated in either the high- or low-risk cohort.

Together, GSVA and GSEA provided complementary evidence on pathway alterations associated with the prognostic model.

### 2.9 Assessment of immune infiltration and function

To characterize the tumor microenvironment (TME) across risk groups, the ESTIMATE algorithm was first applied to derive stromal scores, immune scores, and tumor purity for each sample. Immune infiltration was quantified using the ssGSEA approach, in which curated immune cell–related gene sets were used to calculate enrichment scores for various immune populations, including T cells, NK cells, dendritic cells, and macrophages. Comparisons between high- and low-risk groups were then performed to uncover immune contexture differences associated with the prognostic model. In addition, immune function–related signatures (e.g., antigen presentation, chemokine receptor pathways, cytolytic activity, inflammatory response, and type I/II interferon signaling) were evaluated by ssGSEA to estimate functional activity scores. Statistical testing was used to assess functional discrepancies between groups. Finally, the expression of immune checkpoints and other immune modulatory genes was examined, providing further insights into the association between risk stratification and potential immunotherapeutic responsiveness.

### 2.10 Drug response prediction using GDSC2

To investigate the potential therapeutic implications of the prognostic model, drug response prediction was performed using the Genomics of Drug Sensitivity in Cancer (GDSC2) database (35). Transcriptomic profiles of tumor samples were integrated with pharmacogenomic data to estimate the half-maximal inhibitory concentration (IC50) for a panel of anticancer agents. IC50 represents the drug concentration required to suppress 50% of cell proliferation *in vitro*, with lower values generally indicating higher drug sensitivity. Patients were subsequently stratified into high- and low-risk groups, and differences in predicted drug responses were compared. This analysis provided insight into the association between risk classification and potential treatment responsiveness.

### 2.11 Culture and knockdown experiments of NHA, SF-295, and HS-683

Glioblastoma cell lines SF-295 and HS-683 were purchased from the Cell Bank of the Chinese Academy of Sciences (Shanghai, CBTCCCAS). All cells were cultured in high-glucose DMEM supplemented with 10% fetal bovine serum (FBS) and 1% penicillin–streptomycin, under standard conditions of 37 °C and 5% CO_2_. For gene silencing, siRNAs targeting the candidate gene (sequences listed in **Supplementary Table 2**) were transfected into cells at 50–60% confluence using a commercial reagent according to the manufacturer’s protocol. Cells were incubated for 24–48 h post-transfection, and knockdown efficiency was verified by qRT-PCR, which measured relative expression of the target gene before and after siRNA treatment.

### 2.12 Proliferation analysis using EdU

Cell proliferation was assessed using a 5-ethynyl-2’-deoxyuridine (EdU) incorporation assay kit purchased from a commercial supplier. Cells were seeded in 24-well plates and incubated until reaching appropriate confluence, after which they were exposed to EdU-containing medium for 2 hours, allowing incorporation into newly synthesized DNA during the S phase. Following incubation, cells were fixed, permeabilized, and stained according to the manufacturer’s protocol. Fluorescence microscopy was used to visualize EdU-positive cells under the channel with an excitation wavelength of 550 nm. Proliferation was quantified as the percentage of EdU-positive cells relative to total nuclei, and compared between experimental and control groups.

### 2.13 Assessment of migratory and invasive capacities

Cell migration and invasion abilities were evaluated using Transwell chamber assays.

For the migration assay, serum-starved cells were resuspended in serum-free medium and seeded into the upper chamber inserts (8 μm pore size) without Matrigel coating. The lower chamber was filled with medium containing 10% FBS as a chemoattractant. After 24 hours of incubation, cells remaining on the upper surface were removed with a cotton swab, while migrated cells on the lower surface were fixed, stained with crystal violet, and counted under a microscope in five randomly selected fields.

For the invasion assay, the procedure was similar except that the upper chamber membrane was pre-coated with Matrigel to mimic the extracellular matrix barrier. After 24–48 hours of incubation, cells that invaded through the Matrigel to the lower surface were fixed, stained, and quantified. Migration and invasion capacities were expressed as the average number of cells per field.

### 2.14 Statistical analysis

Two-sided tests were used with P < 0.05 considered significant unless stated otherwise, and multiple testing was controlled by the Benjamini–Hochberg false discovery rate (FDR). Group comparisons used Student’s t-test or Welch’s t-test (parametric) and Wilcoxon rank-sum test (non-parametric) for two groups, and one-way ANOVA or Kruskal–Wallis test for multiple groups. Associations were assessed by Pearson or Spearman correlation as appropriate. Categorical variables were compared by Chi-square or Fisher’s exact test. Survival analyses included Kaplan–Meier curves with log-rank tests and Cox proportional hazards models (HR and 95% CI; proportional-hazards assumption checked by Schoenfeld residuals). Model performance was evaluated by time-dependent ROC/AUC and Harrell’s C-index, with bootstrap resampling for internal validation and calibration where applicable.

## 3 Results

### 3.1 Identification and functional profiling of single-cell populations

**Figure 1:**
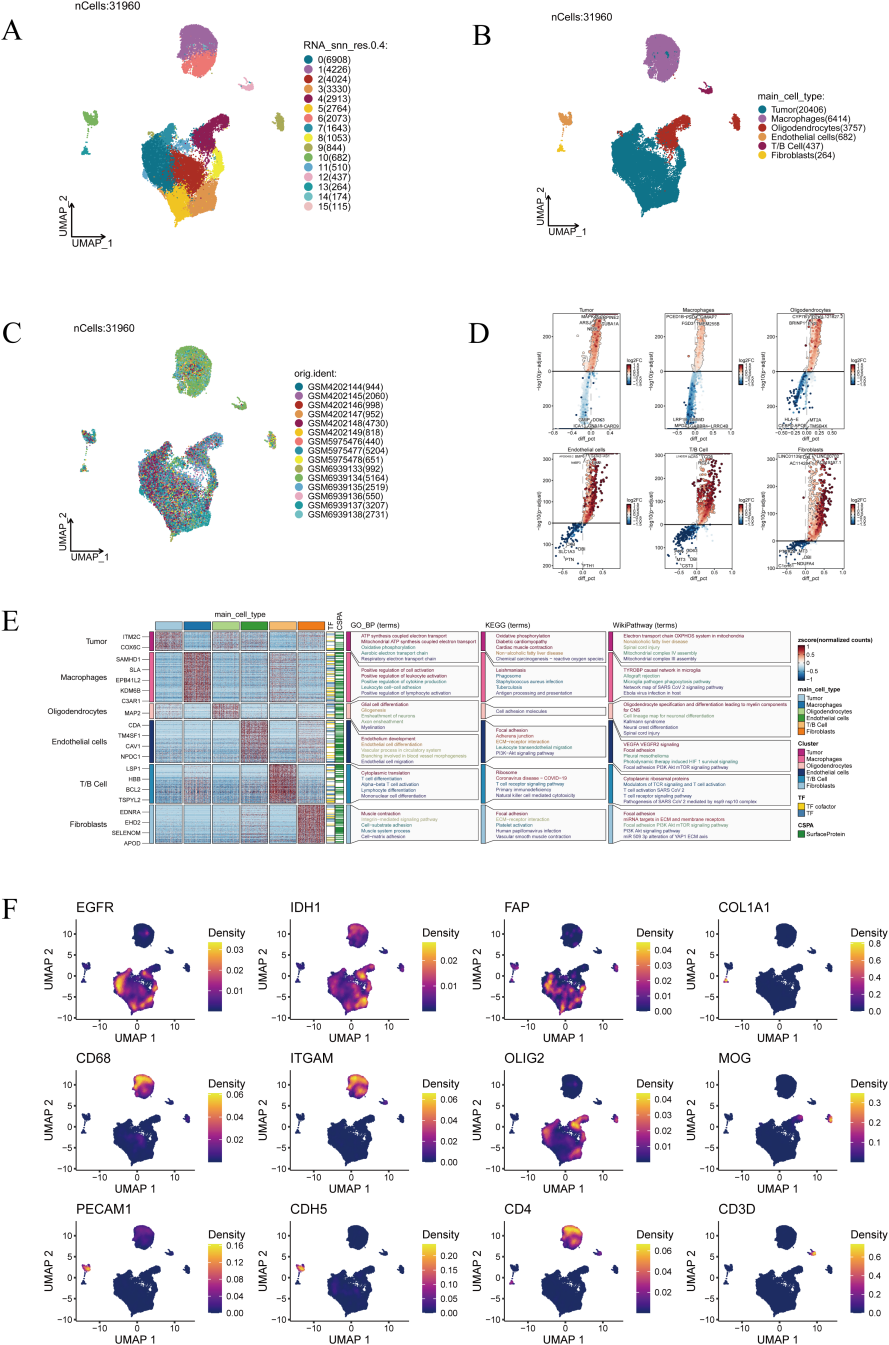
Single-cell transcriptomic landscape of glioblastoma. **(A)** Clustering results of 31,960 cells, grouped into 16 clusters. **(B)** Annotation of major cell types, including tumor cells, macrophages, oligodendrocytes, endothelial cells, T/B cells, and fibroblasts. **(C)** Distribution of cells across different patient samples. **(D)** Visualization of differentially expressed genes across cell clusters. **(E)** Functional enrichment analysis of cluster-specific marker genes. **(F)** Expression patterns of representative marker genes (EGFR, IDH1, FAP, COL1A1, CD68, ITGAM, OLIG2, MOG, PECAM1, CDH5, CD4, and CD3D).

Comprehensive single-cell transcriptomic analysis yielded 31,960 cells in total. UMAP projection revealed 16 well-separated clusters, underscoring the remarkable cellular heterogeneity within GBM (**Figure 1A**). Based on canonical marker expression, these clusters were annotated as tumor cells, macrophages, oligodendrocytes, endothelial cells, T/B lymphocytes, and fibroblasts (**Figure 1B**), reflecting the intricate cellular ecosystem of glioblastoma. When cells from different samples were mapped into the same low-dimensional space, they exhibited an even distribution without clear sample-specific aggregation, indicating that the Harmony algorithm effectively minimized batch effects and ensured robust cross-sample integration (**Figure 1C**). This integrative quality provided a solid foundation for downstream analyses. Differentially expressed gene analysis further delineated the transcriptional identities of each population (**Figure 1D**). Notably, enrichment analysis highlighted distinct biological programs: tumor cells were enriched in oxidative phosphorylation and ATP synthesis coupled electron transport, suggesting enhanced metabolic activity; macrophages showed enrichment in positive regulation of cell activation and leukocyte cell–cell adhesion, pointing to their role in immune regulation and intercellular communication; oligodendrocytes were enriched in glial cell differentiation and cell adhesion molecules, consistent with their function in neural development and myelination (**Figure 1E**). Finally, representative marker genes provided additional validation of the annotation (**Figure 1F**). EGFR and IDH1 were highly expressed in tumor cells, FAP and COL1A1 were confined to fibroblasts, CD68 and ITGAM marked macrophages, PECAM1 and CDH5 identified endothelial cells, OLIG2 and MOG were specific to oligodendrocytes, while CD4 and CD3D defined T-cell subsets. Together, these marker distributions and functional enrichments converged to confirm the biological accuracy of our clustering strategy.

### 3.2 Evaluation of pyroptosis activity at the single-cell level

To comprehensively evaluate the activity of the pyroptosis-related gene set across cell types, five independent scoring methods—AUCell, UCell, singscore, ssGSEA, and AddModuleScore—were applied. The bubble plot revealed consistent patterns across methods: macrophages and T/B cells displayed the highest pyroptosis activity, tumor, endothelial cells, and oligodendrocytes exhibited the lowest, and fibroblasts showed intermediate levels (**Supplementary Figure 1A**). When integrating the results into an average score, the UMAP projection demonstrated clear spatial heterogeneity of pyroptosis activity, with specific tumor cell clusters exhibiting notably elevated scores (**Supplementary Figure 1B**). Violin plot comparisons further confirmed that pyroptosis activity was significantly higher in tumor cells and macrophages compared to other cell types, suggesting that these populations may play pivotal roles in pyroptosis-associated signaling (**Supplementary Figure 1C**). Collectively, these findings validate the robustness of the multi-method scoring approach and establish a reliable foundation for subsequent functional analyses.

### 3.3 Pyroptosis-based stratification reveals remodeling of cell–cell communication

Based on the integrated scoring from five algorithms, all cells were stratified into pyroptosis-high and pyroptosis-low groups for communication network analysis.

**Figure 2:**
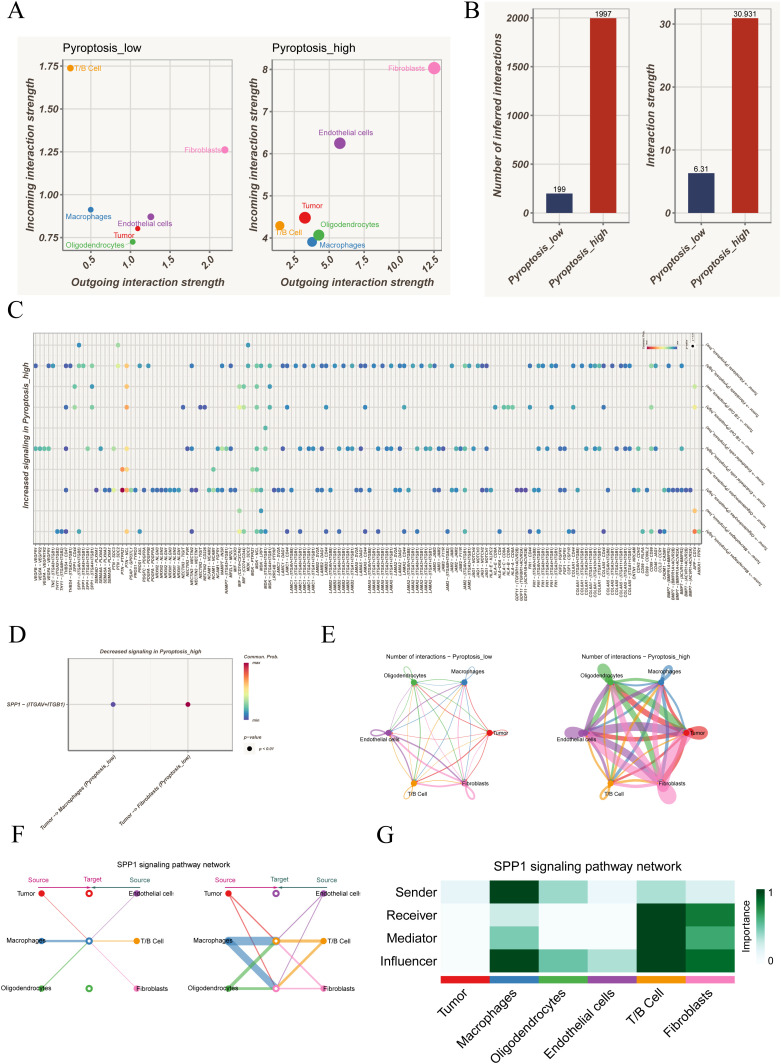
Cell–cell communication analysis under different pyroptosis activity states. **(A)** Distribution of outgoing and incoming signaling strength among cell types in pyroptosis-low versus pyroptosis-high groups, highlighting the distinct roles of each population in the communication network. **(B)** Comparison of the total number and overall strength of intercellular communications between groups, indicating markedly enhanced interactions in the pyroptosis-high state. **(C)** Ligand–receptor pairs significantly upregulated in the pyroptosis-high group, involving multiple immune-related and tumor-associated signaling pathways. **(D)** Ligand–receptor interactions showing reduced activity in the pyroptosis-high group. **(E)** Circle plots depicting intercellular communication networks, contrasting the interaction patterns between pyroptosis-low and pyroptosis-high groups. **(F)** Representative SPP1 signaling network, illustrating the source–target relationships among different cell types. **(G)** Role analysis of the SPP1 pathway across cell populations, showing their functions as signal senders, receivers, mediators, or key influencers.

In the pyroptosis-low group, T/B cells primarily functioned as incoming receivers, while tumor cells and oligodendrocytes acted mainly as outgoing signal senders. With elevated pyroptosis activity, fibroblasts and endothelial cells further enhanced their dual roles in sending and receiving, suggesting substantial remodeling of the communication landscape (**Figure 2A**). Quantitative analysis confirmed that both the total number of interactions and overall communication strength were significantly higher in the pyroptosis-high group (**Figure 2B**). At the ligand–receptor level, several SPP1-associated axes were strengthened in the high group, including SPP1–CD44, SPP1–(ITGAV+ITGB5), SPP1–(ITGA8+ITGB1), SPP1–(ITGA5+ITGB1), and SPP1–(ITGA4+ITGB1) (**Figure 2C**), while SPP1–(ITGAV+ITGB1) was weakened (**Figure 2D**). Given that SPP1 was involved in both strengthened and diminished interactions, a more detailed analysis was performed. Circle plot comparisons revealed denser intercellular communication in the pyroptosis-high group, particularly increased crosstalk between tumor cells and immune populations such as macrophages and T/B cells, with fibroblasts and endothelial cells gaining additional importance (**Figure 2E**). Focusing on the SPP1 pathway, its network visualization highlighted specific interaction directions among cell populations, with macrophages and T/B cells as dominant contributors, while tumor cells played a limited role (**Figure 2F**). Role analysis further demonstrated that macrophages mainly functioned as Senders and Influencers, whereas T/B cells exhibited multiple roles as Receivers, Mediators, and Influencers; in contrast, tumor cells showed minimal involvement (**Figure 2G**). Collectively, these results indicate that the SPP1 pathway is strongly activated under heightened pyroptosis activity, predominantly orchestrated by immune cells.

### 3.4 Reclustering highlights functional heterogeneity of malignant epithelial cells

**Figure 3:**
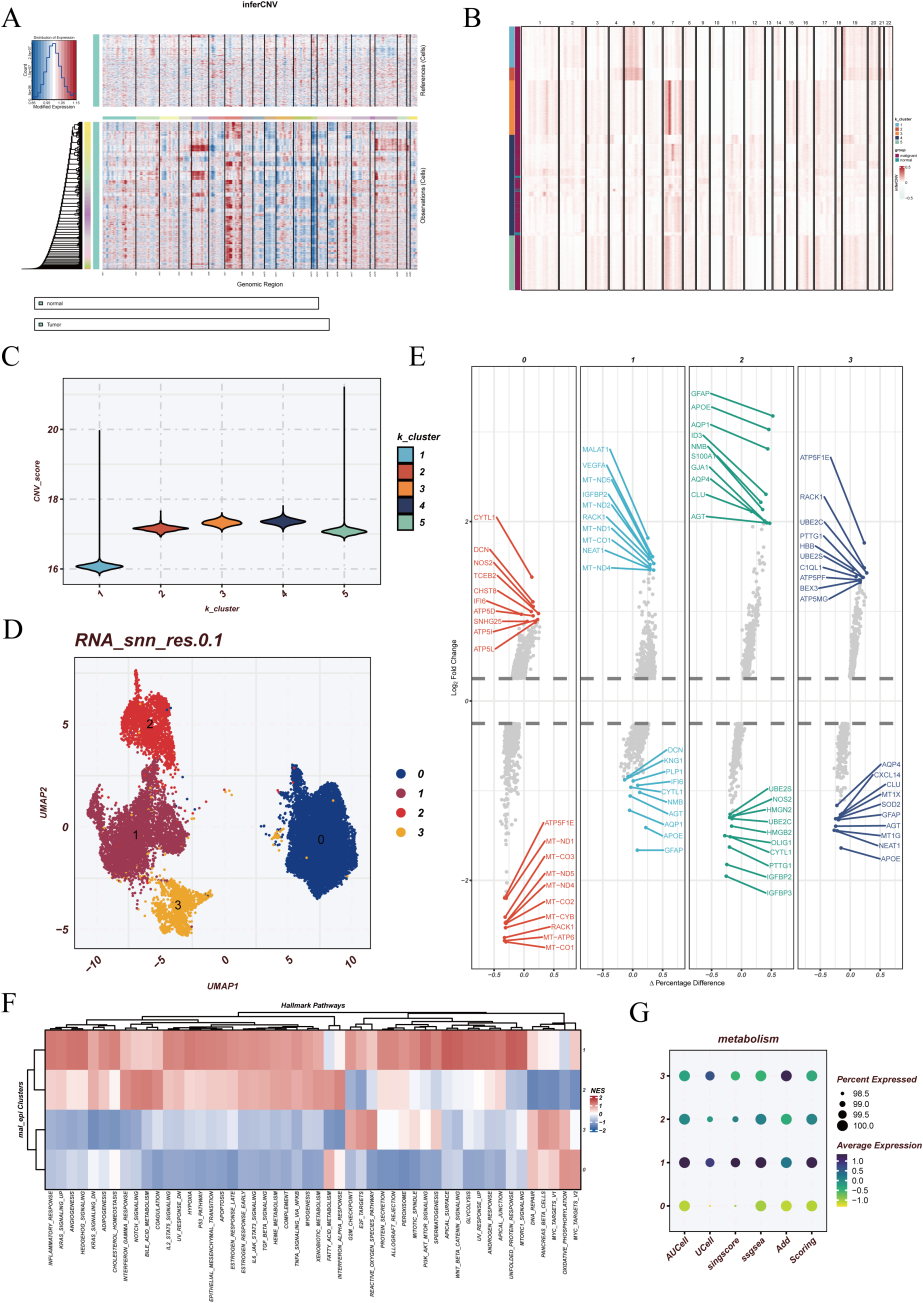
Identification and reclustering of malignant epithelial cells. **(A)** Results of inferCNV analysis using endothelial cells as the reference population. The upper panel represents reference cells with relatively stable copy number patterns, while the lower panel shows tumor cells with extensive chromosomal alterations, indicating malignant properties. **(B)** k-means clustering based on the inferCNV expression matrix, where distinct colors represent different CNV-defined cell populations. **(C)** Violin plots showing the distribution of CNV scores across each malignant cluster. **(D)** UMAP visualization of reclustered malignant epithelial cells, identifying four major subclusters. **(E)** Differentially expressed genes (DEGs) across the four subclusters. **(F)** Functional enrichment analysis using HALLMARK gene sets, highlighting distinct biological processes in each malignant subcluster. **(G)** Pyroptosis enrichment scores of malignant cells calculated using five different algorithms: AUCell, UCell, singscore, ssGSEA, and AddModuleScore.

In the single-cell landscape of GBM, epithelial cells represent the primary tumor-derived population. However, not all epithelial cells display uniform malignant features, as some may resemble normal counterparts. To distinguish malignant from non-malignant populations, inferCNV analysis was performed using endothelial cells as the reference. The results revealed widespread chromosomal copy number variations across epithelial cells, while reference cells maintained stable profiles, indicating pronounced tumor-associated genomic alterations (**Figure 3A**). Subsequent k-means clustering divided the cells into five groups, with clusters 2, 3, 4, and 5 showing elevated CNV burdens and classified as malignant epithelial cells, whereas cluster 1 was closer to the reference profile (**Figures 3B–C**).

Reclustering of the malignant epithelial cells identified four distinct subclusters (clusters 0, 1, 2, and 3) in UMAP visualization (**Figure 3D**). Differential expression analysis highlighted unique transcriptional programs in each subgroup (**Figure 3E**). Functional enrichment based on HALLMARK gene sets suggested that cluster 1 was broadly correlated with multiple hallmark pathways, reflecting a globally activated state. Cluster 0 was associated with OXIDATIVE_PHOSPHORYLATION, MYC_TARGETS_V2, and FATTY_ACID_METABOLISM, implicating metabolic reprogramming and energy supply in supporting rapid tumor proliferation. Cluster 3 was enriched in G2M_CHECKPOINT and REACTIVE_OXYGEN_SPECIES_PATHWAY, suggesting potential roles in cell cycle regulation and oxidative stress responses (**Figure 3F**). These findings indicate that malignant epithelial cells, despite sharing tumor origin, display functional divergence that may represent different states of tumor progression.

Furthermore, pyroptosis enrichment analysis revealed distinct heterogeneity across the malignant subclusters. Cluster 1 exhibited the highest pyroptosis activity, whereas cluster 0 showed the lowest, with other clusters displaying intermediate levels (**Figure 3G**). This highlights the diverse regulation of pyroptosis among malignant populations, adding another layer of functional heterogeneity in GBM epithelial cells.

### 3.5 Pseudotime trajectories and transcriptional regulatory features of malignant epithelial cells

**Figure 4:**
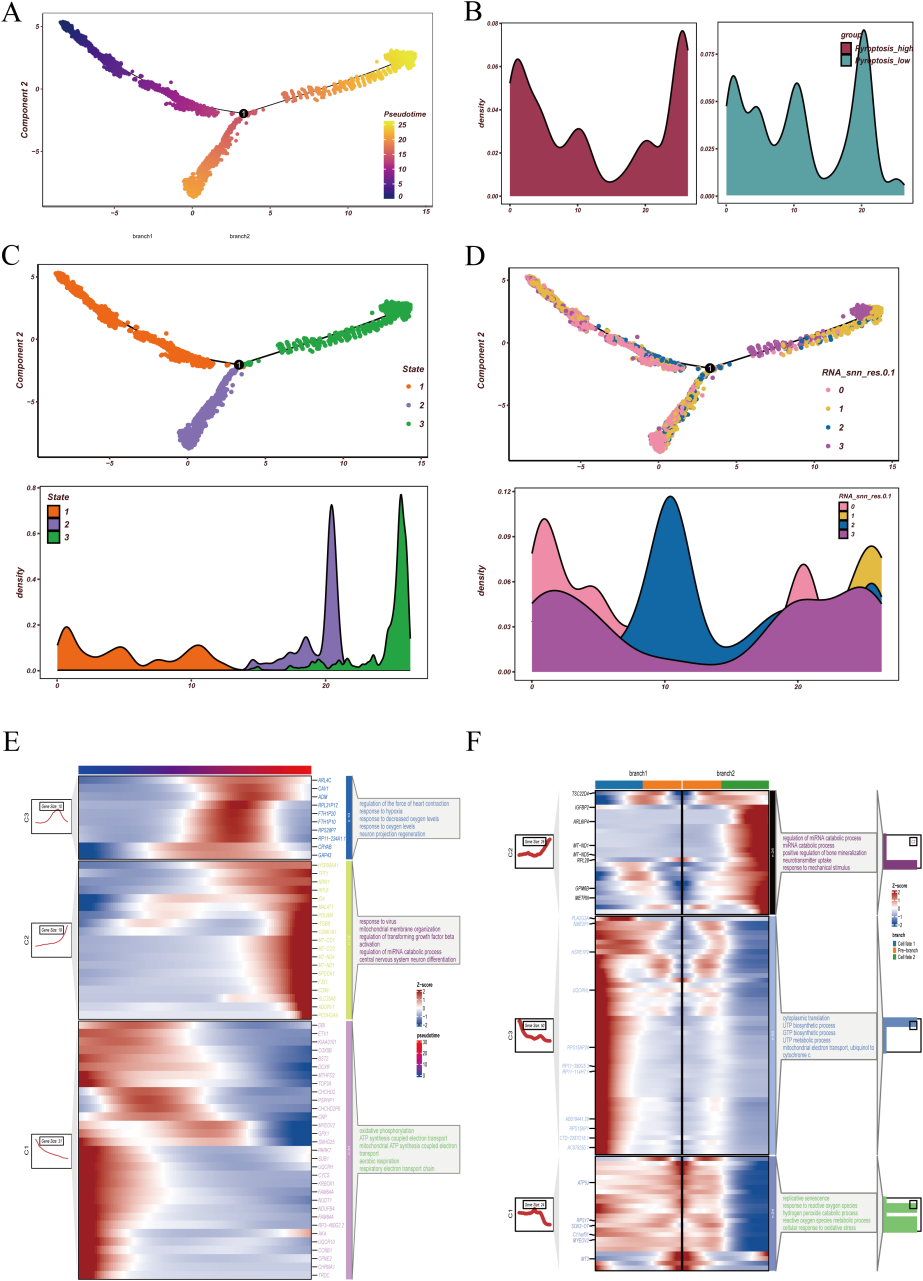
Pseudotime trajectory and differentiation branches of malignant epithelial cells. **(A)** Pseudotime analysis of malignant epithelial cells, with distinct colors indicating cells at different pseudotime states. **(B)** Distribution of high- and low-pyroptosis activity groups along the pseudotime trajectory, highlighting divergent patterns across differentiation paths. **(C)** State assignment of malignant cells. The upper panel shows pseudotime trajectory colored by states, and the lower panel displays the density distribution of cells within each state. **(D)** Projection of RNA_snn_res.0.1 clusters onto the pseudotime trajectory. The upper panel depicts the spatial distribution of clusters, while the lower panel illustrates density profiles of each cluster across pseudotime. **(E)** Dynamic expression trends of representative genes during pseudotime progression, accompanied by functional enrichment analyses, demonstrating sequential transcriptional changes along differentiation. **(F)** Functional enrichment analyses across divergent pseudotime branches. At the bifurcation point originating from state 1, distinct branches were enriched in pathways associated with specific biological processes, suggesting different functional fates of malignant epithelial cells during differentiation.

To further characterize the dynamic evolution of malignant epithelial cells, we first applied Monocle2 for pseudotime analysis (**Figure 4**). The trajectory heatmap (**Figure 4A**) demonstrated a continuous distribution of malignant clusters across pseudotime, indicating a progressive differentiation pattern. When examining pyroptosis activity (**Figure 4B**), we observed that cells with high pyroptosis activity were predominantly located at both the beginning and the end of the trajectory, whereas low-activity cells were enriched in the middle phase, suggesting a potential role of pyroptosis in both initiation and terminal differentiation.

Overall, cells were divided into three states: state 1 represented early differentiation, while states 2 and 3 corresponded to distinct late-stage branches (**Figure 4C**). Reclustering revealed that cluster 2 was largely distributed in the intermediate stage, whereas clusters 0, 1, and 3 were detected at both the beginning and the endpoints of the trajectory (**Figure 4D**). Regarding gene expression dynamics, genes upregulated along pseudotime were enriched in hypoxia-related and oxygen-response pathways, while downregulated genes were mainly involved in mitochondrial ATP synthesis coupled electron transport (**Figure 4E**). Branch analysis further showed that branch 2 was associated with miRNA catabolism and bone mineralization regulation, whereas branch 1 was enriched in pyrimidine and purine biosynthesis (**Figure 4F**). Collectively, these findings suggest that GBM malignant epithelial cells undergo metabolic and stress-related remodeling during differentiation and display distinct functional programs along different branches.

To validate and complement these observations, we next reconstructed trajectories using the Slingshot algorithm. Two major lineages were identified (**Supplementary Figure 2A**). In the UMAP embedding, cluster 0 cells were positioned at the beginning of the trajectory, cluster 1 cells at the intermediate stage, while clusters 2 and 3 occupied the terminal branches (**Supplementary Figure 2B**). Functional enrichment analysis revealed that Lineage 1 (cluster 0 → 1 → 2) was associated with regulation of trans-synaptic signaling, long-term synaptic potentiation, and glucocorticoid responses, while the other lineage showed distinct functional orientations (**Supplementary Figure 2C**). At the regulatory level, SCENIC analysis indicated that cluster 0 was strongly linked to transcription factors such as SIX5, E2F6, POLR3G, and TP53 (**Supplementary Figure 2D**), highlighting their potential roles in early differentiation and cell fate specification.

In summary, these findings depict both the temporal progression and branch-specific divergence of GBM malignant epithelial cells, underscoring the importance of transcriptional networks in orchestrating early-stage transitions.

### 3.6 Development of a pyroptosis-related gene signature for risk stratification and therapeutic prediction

Before model construction, differentially expressed genes between malignant epithelial cells with distinct pyroptosis activity levels were identified and subjected to univariate Cox regression. A considerable number of these genes were significantly associated with overall survival (**Supplementary Figure 3A**). PCA across the CGGA, GSE13041, GSE74187, GSE83300, and TCGA-GBM cohorts revealed substantial batch effects prior to correction (**Supplementary Figure 3B**). After adjustment with the SVA algorithm, the distribution of samples became more homogeneous across datasets (**Supplementary Figure 3C**), confirming that batch effects were effectively removed and that the integrated dataset was suitable for downstream modeling.

**Figure 5:**
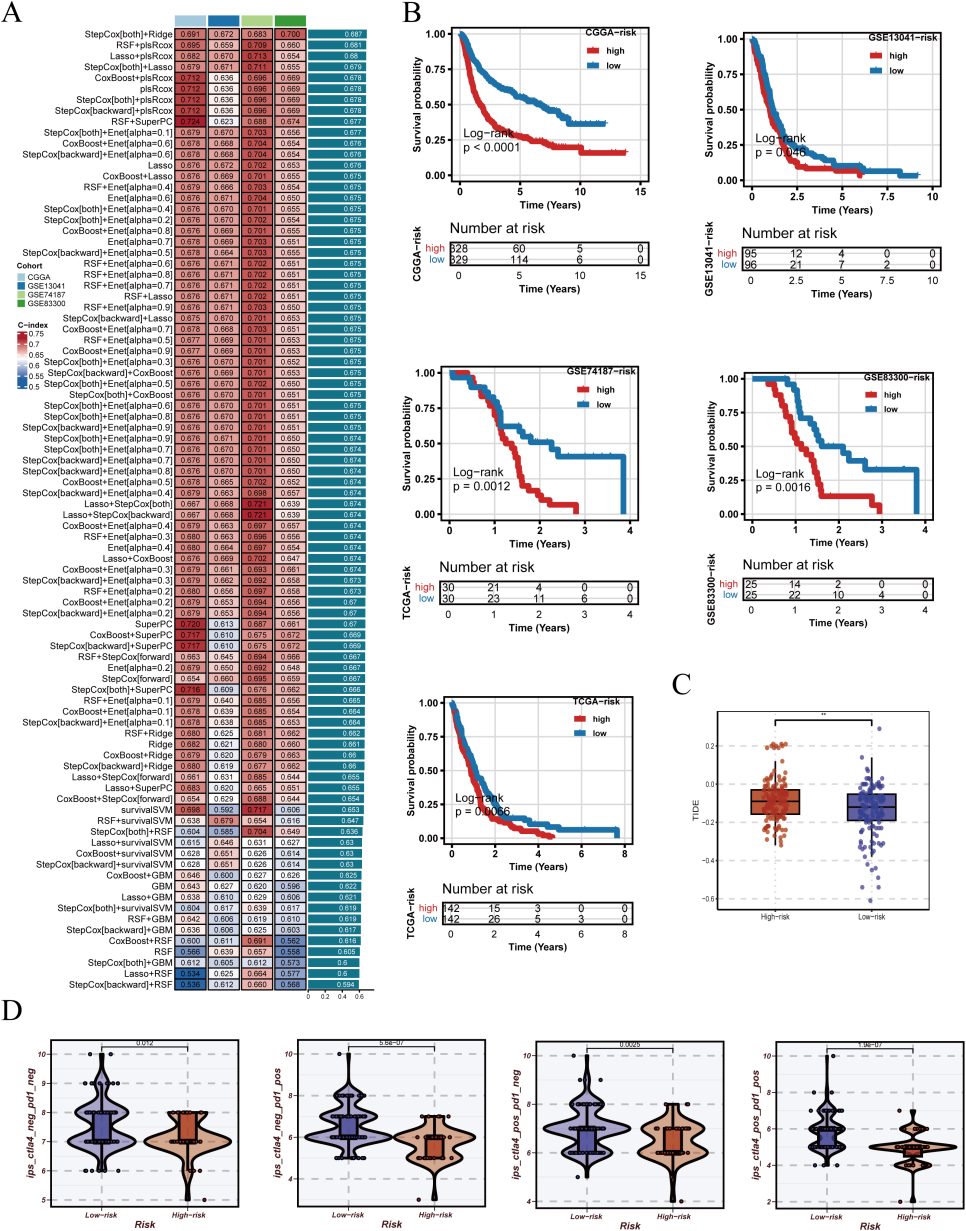
Construction of PRGS and prediction of immunotherapy response. **(A)** PRGS was developed based on 10 machine-learning algorithms and their combinations, ranked by C-index in the training set. **(B)** Patients were stratified into groups according to the median PRGS risk score, and Kaplan–Meier survival curves are displayed. **(C)** Distribution of TIDE scores across different PRGS risk groups. **(D)** Distribution of immunophenoscores (IPS) across different PRGS risk groups.

Based on the adjusted data, ten machine learning algorithms and their combinations were systematically applied to construct prognostic models, with performance ranked by C-index (**Figure 5A**). After identifying StepCox[both]+Ridge as the optimal modeling approach, we further examined the expression patterns of PRGS component genes in the integrated GBM single-cell dataset (**Supplementary Figure 4**). These genes displayed distinct expression distributions across cellular populations, with several showing elevated expression in malignant epithelial cells. This observation not only supports the biological plausibility of PRGS from a single-cell perspective but also provides additional evidence for the potential roles of these genes in glioblastoma progression. The optimal strategy was identified as StepCox[both]+Ridge, which was subsequently used to establish the Pyroptosis-Related Gene Signature (PRGS). Patients were stratified into high- and low-risk groups using the median PRGS score, and Kaplan–Meier analysis revealed significantly worse survival in the high PRGS group (**Figure 5B**). Immunological evaluation further demonstrated that high-risk patients exhibited elevated TIDE scores (**Figure 5C**), suggesting greater immune evasion potential. In addition, across four distinct immunogenic states, IPS comparisons consistently indicated lower scores in the low-risk group, implying a higher likelihood of benefiting from immunotherapy (**Figure 5D**).

**Figure 6:**
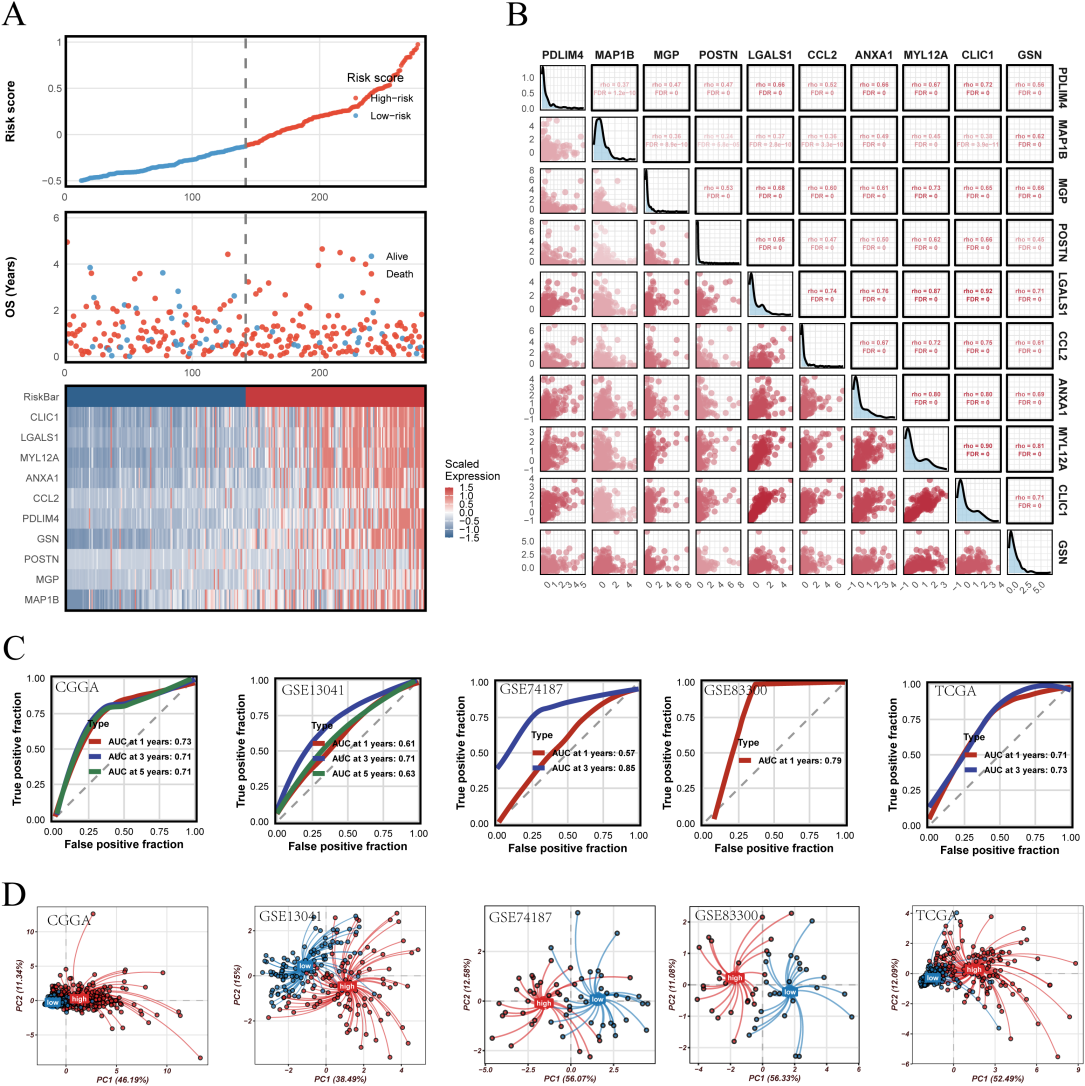
Validation of the PRGS and prognostic stratification. **(A)** Distribution of risk scores, overall survival status, and the corresponding heatmap of PRGS gene expression in glioblastoma patients. **(B)** Pairwise correlation analysis among the genes included in the PRGS model. **(C)** Time-dependent ROC curves evaluating the predictive performance of the PRGS at 1-, 3-, and 5-year survival across multiple cohorts. **(D)** Principal component analysis (PCA) plots illustrating the separation between high- and low-PRGS groups in independent datasets.

The robustness of PRGS was then assessed across multiple independent validation cohorts. Risk curves, survival distributions, and heatmaps of gene expression consistently indicated unfavorable outcomes in the high-risk group (**Figure 6A**). Most PRGS genes were positively correlated, with the strongest correlation observed between MYL12A and MGP (rho = 0.73, FDR = 0) (**Figure 6B**). ROC analysis demonstrated that PRGS maintained strong predictive performance at 1-, 3-, and 5-year survival endpoints across diverse cohorts (**Figure 6C**). PCA plots further confirmed that PRGS clearly separated high- and low-risk patients across datasets (**Figure 6D**). Taken together, these findings highlight PRGS as a robust and generalizable prognostic signature with potential utility in guiding risk stratification and predicting immunotherapy response in GBM.

### 3.7 Metabolic and immune pathway differences driven by PRGS stratification

**Figure 7:**
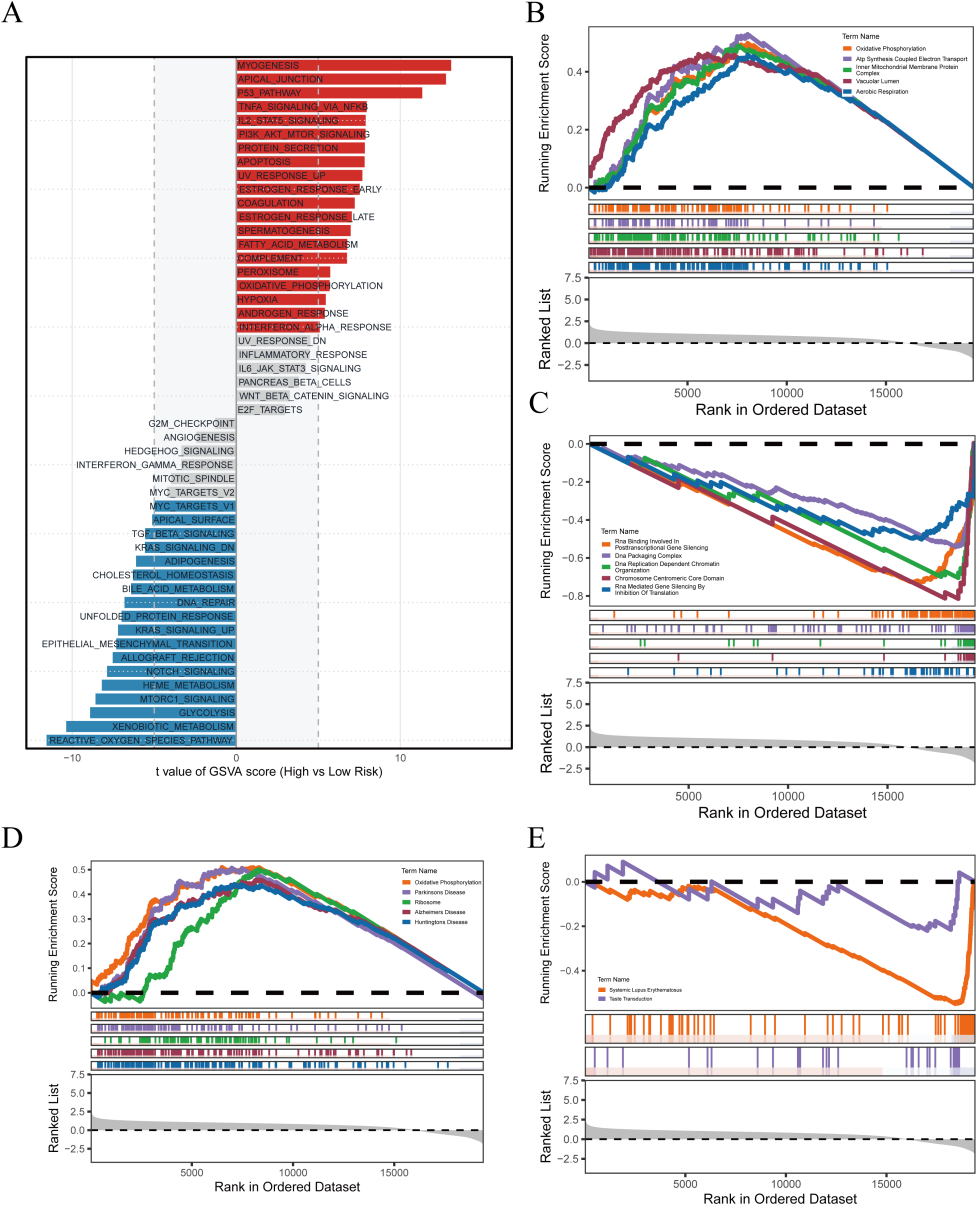
Pathway enrichment associated with PRGS risk stratification. **(A)** GSVA delineated global functional differences across PRGS subgroups. **(B)** High-PRGS cells were linked to specific GO processes identified through GSEA. **(C)** Low-PRGS cells were connected with alternative GO processes detected by GSEA. **(D)** KEGG-based enrichment analysis indicated pathways characteristic of the high-PRGS population. **(E)** Distinct KEGG programs were identified in the low-PRGS population.

In the preceding analyses, PRGS was shown to hold significant value for prognostic prediction and immunotherapy response assessment. To further elucidate the biological basis underlying these findings, we conducted functional enrichment analyses to compare transcriptional characteristics between the high- and low-risk groups. The GSVA results (**Figure 7A**) demonstrated that the high-PRGS group was enriched in pathways such as MYOGENESIS, APICAL_JUNCTION, and P53_PATHWAY, whereas the low-PRGS group was predominantly enriched in REACTIVE_OXYGEN_SPECIES_PATHWAY, XENOBIOTIC_METABOLISM, and GLYCOLYSIS, highlighting divergent stress and metabolic processes. GO-based GSEA (**Figures 7B, C**) further revealed that the high-PRGS group exhibited strong associations with energy metabolism pathways, including Oxidative Phosphorylation and ATP Synthesis Coupled Electron Transport, while the low-PRGS group was more closely linked to RNA regulatory functions such as RNA Binding Involved in Posttranscriptional Gene Silencing. KEGG enrichment (**Figures 7D, E**) indicated that high-PRGS tumors were primarily associated with Parkinson’s Disease and Ribosome, whereas the low-PRGS group was enriched in immune- and signaling-related pathways, including Systemic Lupus Erythematosus and Taste Transduction.

Taken together, these analyses highlight the multidimensional molecular characteristics reflected by PRGS stratification: tumors with high PRGS scores tend to exhibit greater metabolic demands and stress adaptation, while those with low PRGS scores rely more heavily on metabolic flexibility and immune-related processes. These distinctions not only provide mechanistic support for the predictive capacity of PRGS but also suggest its potential role in driving metabolic reprogramming, shaping the tumor immune microenvironment, and modulating therapeutic sensitivity, thereby offering a biological rationale for future translational research and precision treatment strategies in GBM.

### 3.8 Systematic evaluation of PRGS in the tumor microenvironment and drug response

To further elucidate the role of the PRGS in shaping the tumor microenvironment and influencing therapeutic responses, we performed a comprehensive analysis across different risk groups.

**Figure 8:**
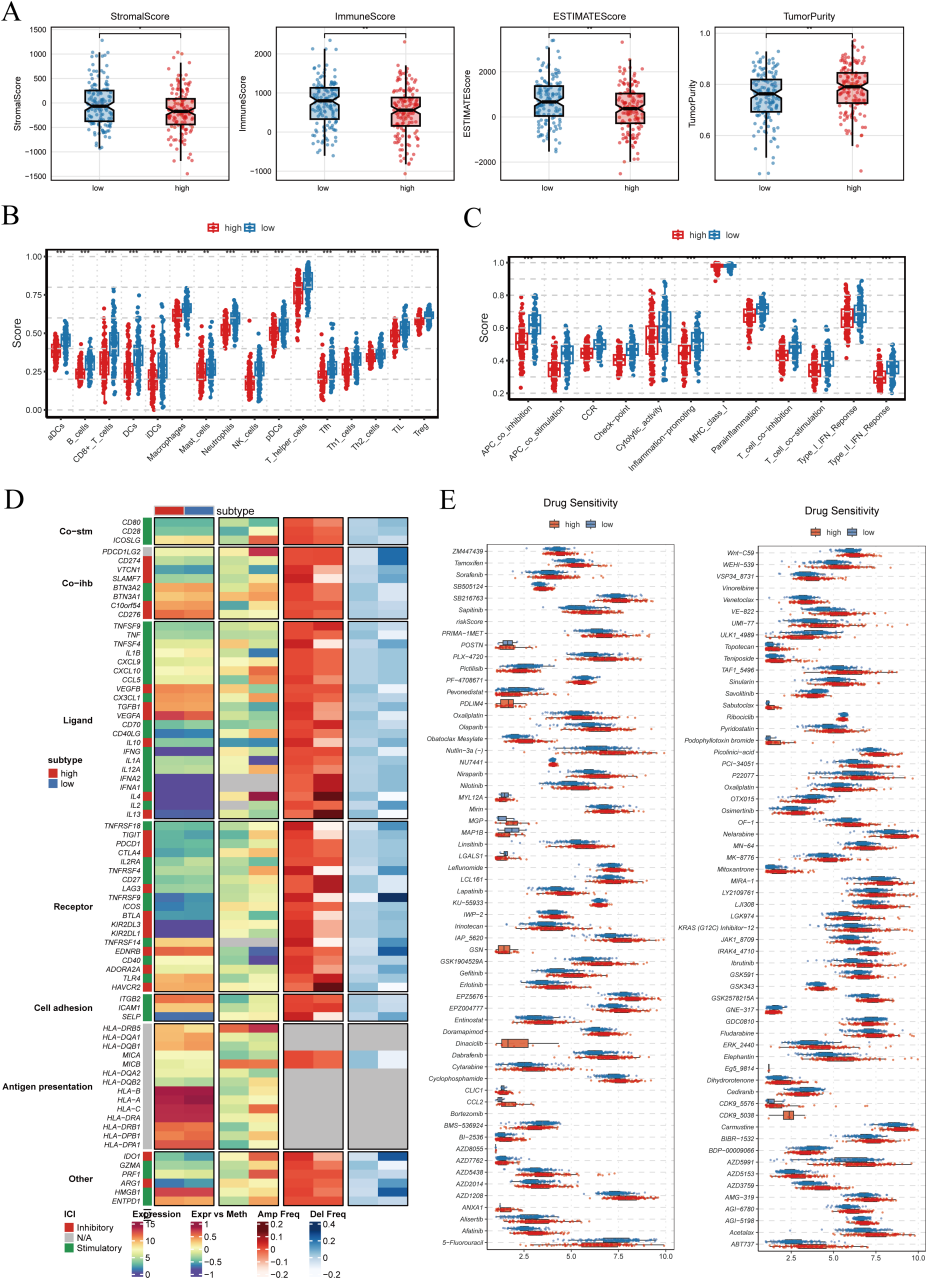
Tumor microenvironment characteristics and drug sensitivity analysis. **(A)** StromalScore, ImmuneScore, ESTIMATEScore, and TumorPurity were calculated between high- and low-PRGS groups using the ESTIMATE algorithm. **(B)** Differences in immune cell infiltration levels between high- and low-PRGS groups. **(C)** Comparison of immune-related functional activities across PRGS subgroups. **(D)** Immune modulators showing distinct patterns between PRGS groups, including co-stimulatory molecules (Co-stim), co-inhibitory molecules (Co-inh), ligands, receptors, cell adhesion molecules, and antigen presentation-related factors, integrating multi-omics information such as gene expression, DNA methylation, and copy number alterations (amplification/deletion frequency). **(E)** Predicted drug sensitivity analysis based on the GDSC2 database, evaluating differences in half-maximal inhibitory concentration (IC50) between high- and low-PRGS groups, where lower IC50 values indicate greater drug sensitivity.

As shown in **Figure 8A**, the ESTIMATE algorithm revealed that patients in the high-PRGS group exhibited higher tumor purity, while StromalScore, ImmuneScore, and the overall ESTIMATEScore were comparatively lower. This indicates that non-tumor components account for a smaller fraction of the tumor tissue in the high-risk group. The analysis of immune cell infiltration demonstrated that the low-PRGS group displayed higher levels of infiltration across multiple immune cell subsets (**Figure 8B**). Consistently, immune-related functional activities, including antigen presentation, cytolytic activity, and inflammatory responses, were more active in the low-risk group, whereas these processes were relatively weaker in the high-risk group (**Figure 8C**). These findings suggest that the low-PRGS group may be characterized by enhanced immune surveillance and effector activity. When examining immune regulatory factors, integration of gene expression, methylation, and copy number variation data revealed notable differences between the two groups (**Figure 8D**). These differences involved a wide range of molecules, including immune co-stimulatory and co-inhibitory factors, ligand–receptor interactions, cell adhesion molecules, and antigen-presenting pathways, highlighting the multidimensional association between PRGS and immune regulatory networks.

Drug sensitivity prediction further revealed significant differences in the half-maximal inhibitory concentration (IC50) values of multiple compounds between the two groups (p < 0.001), with representative drugs shown in **Figure 8E**. Since IC50 values are inversely correlated with drug sensitivity, these findings imply that patients with different PRGS levels may respond differentially to specific therapies, underscoring the potential value of PRGS in guiding personalized treatment strategies.

Taken together, these results demonstrate that PRGS is closely associated with tumor purity, immune infiltration, functional immune activity, and drug sensitivity. This not only provides further biological insights into the clinical utility of PRGS but also highlights its promise for precision oncology and immunotherapeutic interventions.

### 3.9 Mutation landscape and genomic alterations associated with PRGS

**Figure 9:**
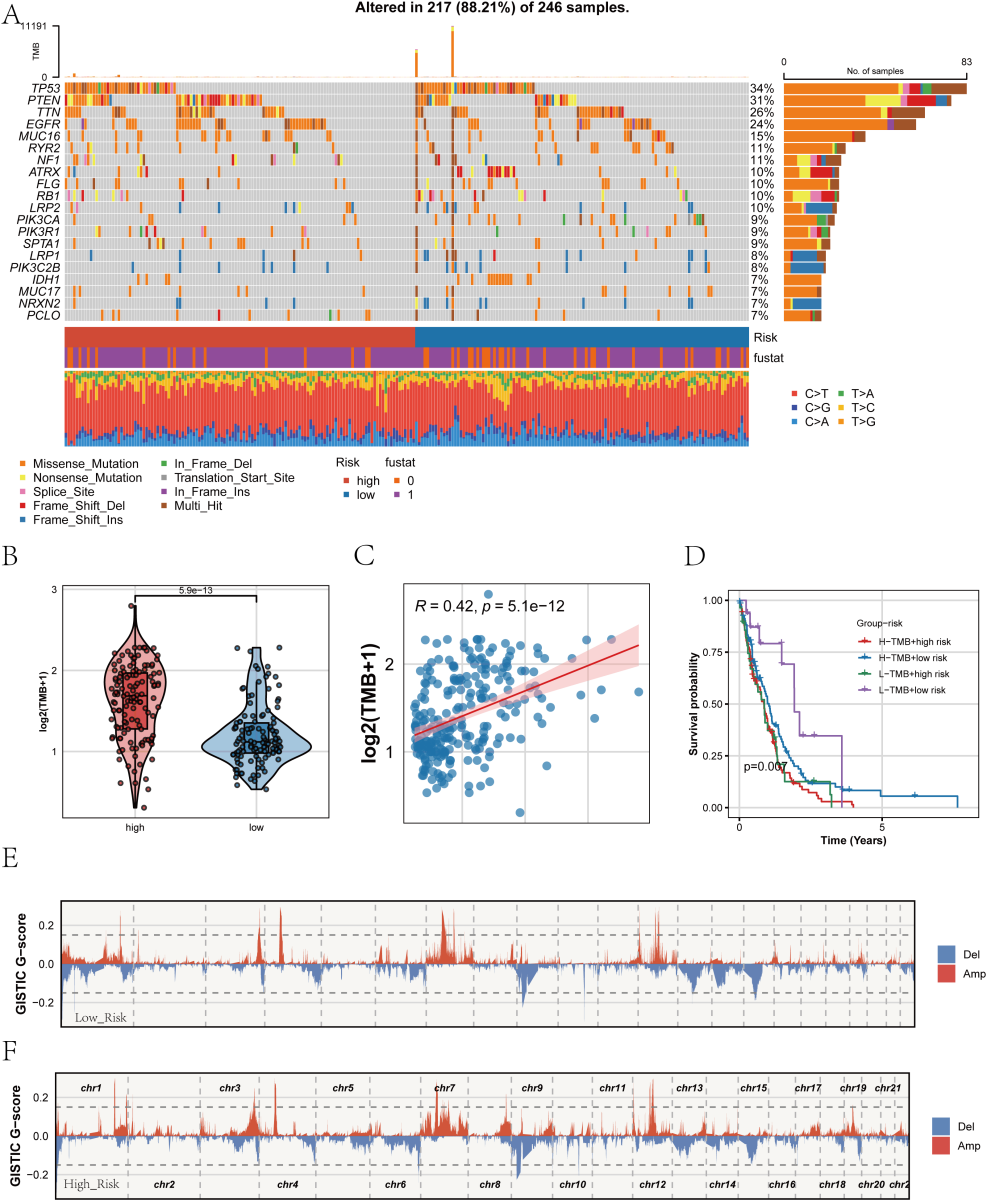
Mutation landscape and genomic alterations associated with PRGS. **(A)** Oncoplot showing the somatic mutation profiles of the high- and low-PRGS groups. The upper panel depicts the overall mutation frequency, while the side bar indicates the proportion of different mutation types across groups. **(B)** Violin plot comparing tumor mutational burden (TMB) levels between high- and low-PRGS groups. **(C)** Scatter plot illustrating the correlation between TMB and PRGS risk score. **(D)** Kaplan–Meier curves of overall survival in patients stratified by both PRGS and TMB subgroups. **(E, F)** Copy number variation (CNV) landscapes inferred by GISTIC2.0, showing chromosomal amplification (red) and deletion (blue) events in low- **(E)** and high-PRGS **(F)** groups.

After establishing the strong association of the PRGS with prognosis and immune characteristics, we next investigated its genomic underpinnings. Comparison of somatic mutation landscapes between the high- and low-PRGS groups revealed widespread genetic alterations across both cohorts, with canonical driver genes such as TP53, PTEN, and EGFR showing high mutation frequencies (**Figure 9A**). These findings suggest that alterations in core oncogenic pathways are central to glioblastoma pathogenesis. We then examined tumor mutational burden (TMB) across PRGS-defined subgroups (**Figure 9B**). The high-PRGS group exhibited significantly elevated TMB compared with the low-PRGS group, indicating greater genomic instability among high-risk patients. Correlation analysis further confirmed a positive association between PRGS scores and TMB (R = 0.42, p < 0.001; **Figure 9C**), reinforcing the capacity of PRGS to reflect the mutational background of tumors. To assess the joint prognostic implications of PRGS and TMB, we performed stratified survival analysis (**Figure 9D**). Patients harboring both high PRGS and high TMB exhibited the worst overall survival, whereas those with low PRGS and low TMB displayed the most favorable outcomes. This highlights the added value of integrating PRGS and TMB for survival stratification in glioblastoma. Finally, copy number variation (CNV) profiles were assessed using GISTIC2.0 (**Figures 9E, F**). Both subgroups demonstrated extensive chromosomal amplifications and deletions; however, the magnitude of CNV alterations was more pronounced in the high-PRGS group, with large-scale aberrations observed in multiple chromosomal regions. These findings suggest that PRGS is closely linked to genomic instability.

Taken together, these analyses indicate that the PRGS not only correlates with clinical outcomes and immune states but also captures the mutational and CNV landscape of glioblastoma, underscoring its potential as a multidimensional biomarker for biological and clinical characterization.

### 3.10 Functional validation of *MAP1B* as an oncogenic driver in GBM cells

As demonstrated in our previous analyses, the PRGS was constructed using multiple machine-learning combinations and validated across independent cohorts, showing robust prognostic value (**Figures 5**, **6**). Among the genes included in the signature, *MAP1B* was prioritized for further functional investigation due to its relatively high hazard ratio in univariate Cox regression and previous reports implicating its role in neural development and tumor progression.

**Figure 10:**
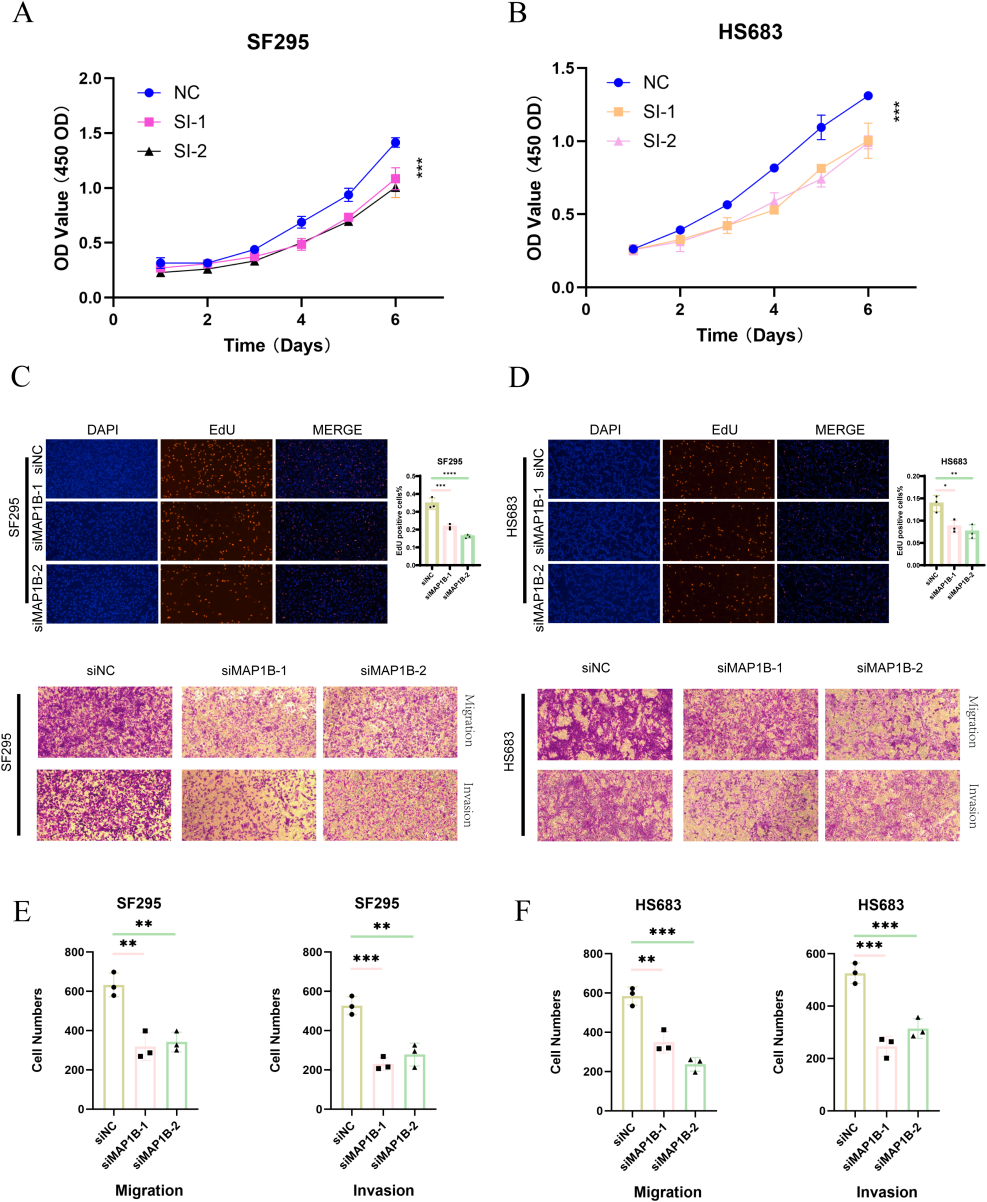
Functional validation of *MAP1B* in glioblastoma cell lines. **(A, B)** CCK-8 assay showing the proliferative capacity of SF295 and HS683 cells after transfection with control siRNA (siNC) or *MAP1B*-targeting siRNAs (si*MAP1B*-1, si*MAP1B*-2). **(C, D)** EdU incorporation assay assessing DNA synthesis in the two cell lines following *MAP1B* knockdown, with DAPI staining used for nuclear visualization. **(E, F)** Transwell assays evaluating the effects of *MAP1B* silencing on cell migration and invasion in SF295 and HS683 cells.

*In vitro* experiments confirmed the oncogenic function of *MAP1B* in GBM cells. CCK-8 assays indicated that *MAP1B* silencing markedly reduced the proliferative capacity of SF295 and HS683 cells (**Figures 10A, B**). EdU incorporation assays further confirmed its contribution to DNA synthesis and proliferation (**Figures 10C, D**). Moreover, Transwell assays demonstrated that *MAP1B* knockdown significantly impaired the migratory and invasive abilities of GBM cells (**Figures 10E, F**).

Together, these findings not only provide experimental validation for the PRGS model but also highlight *MAP1B* as a potential therapeutic target in GBM.

## 4 Discussion

GBM is notorious for its aggressiveness, therapeutic resistance, and devastating clinical outcomes (36). Although standard-of-care regimens combining maximal safe resection, radiotherapy, and temozolomide chemotherapy have modestly extended survival, the median overall survival remains under 18 months (37). More recently, novel approaches such as immune checkpoint inhibitors and tumor-treating fields have been introduced, yet their benefits have been limited to a small subset of patients. The principal challenge lies in the profound intratumoral heterogeneity and adaptive plasticity of GBM, which collectively underlie immune evasion, metabolic reprogramming, and resistance to therapy (38, 39). Against this backdrop, identifying robust prognostic markers and dissecting their biological underpinnings are essential steps toward precision medicine in GBM.

Bulk transcriptome analyses have previously yielded molecular classifications and survival-associated signatures in GBM, but their interpretive power is constrained by the averaging of signals across mixed cell populations (40). scRNA-seq, in contrast, offers a powerful lens to resolve this complexity, enabling the dissection of malignant and non-malignant compartments, cell–cell communication networks, and evolutionary trajectories of tumor cells (41). By integrating scRNA-seq with bulk transcriptomes from multiple large cohorts, our study developed and validated PRGS that not only stratifies GBM patients into distinct risk groups but also reflects immunological and therapeutic vulnerabilities.

Our findings revealed that PRGS reliably predicted overall survival and provided insights into immunotherapy response. Patients with high PRGS scores exhibited poorer prognosis and higher TIDE scores, indicative of enhanced immune escape, whereas low PRGS patients showed consistently lower IPS values across immunogenic states, suggesting greater benefit from immunotherapy. These results underscore the clinical relevance of PRGS as both a prognostic and predictive biomarker. Importantly, scRNA-seq–based communication analysis pinpointed the SPP1 axis as a critical signaling pathway enriched in the high-pyroptosis group. SPP1 (osteopontin), a multifunctional extracellular matrix protein, has been implicated in macrophage recruitment, angiogenesis, and promotion of an immunosuppressive tumor microenvironment in diverse cancers (42–44). In GBM, our analysis indicated that macrophages acted as dominant senders of SPP1 signals, while T/B cells and malignant epithelial cells frequently served as receivers. This pattern suggests that SPP1 may function as a central mediator of immune remodeling in pyroptosis-high tumors, reinforcing tumor-promoting interactions between stromal and immune compartments.

Beyond immune signaling, pseudotime and transcription factor analyses further illuminated how pyroptosis activity intersects with tumor cell differentiation and metabolic programs. Cells with high pyroptosis scores tended to reside at both early and terminal branches of differentiation trajectories, indicating a role in tumor plasticity. Moreover, trajectory-specific enrichment revealed links to hypoxia response, oxidative phosphorylation, and nucleoside biosynthesis, suggesting that pyroptosis may act as a modulator of metabolic state transitions that shape malignant progression.

Among the genes included in PRGS, *MAP1B* emerged as a compelling candidate for functional validation, given its strong association with survival in univariate Cox analysis and supportive evidence from prior studies. *MAP1B* encodes microtubule-associated protein 1B, a key regulator of cytoskeletal dynamics and neuronal development (45). Dysregulated *MAP1B* expression has been reported in breast, colorectal, and brain tumors, where it promotes proliferation, invasion, and therapeutic resistance (46–49). Consistent with these reports, our *in vitro* assays demonstrated that *MAP1B* knockdown suppressed proliferation, DNA synthesis, and invasive capacity in GBM cells, highlighting its oncogenic role. These findings position *MAP1B* not only as a model-derived risk gene but also as a potential therapeutic target whose inhibition may attenuate GBM aggressiveness.

Several limitations of our work warrant discussion. Although PRGS was constructed and validated across multiple independent datasets, prospective clinical cohorts are needed to confirm its predictive utility in real-world settings. Our functional validation focused primarily on *MAP1B*, and additional experimental efforts are required to explore the contributions of other PRGS genes. Moreover, while our integrative analyses identified SPP1- and *MAP1B*-related pathways as potentially central to GBM biology, mechanistic dissection through animal models and molecular experiments will be critical to fully elucidate their roles.

In conclusion, this study introduces PRGS as a robust pyroptosis-related signature with strong prognostic and therapeutic predictive value in GBM. By leveraging scRNA-seq and bulk transcriptomics, we not only provided a clinically relevant biomarker but also revealed mechanistic insights into how pyroptosis intersects with immune remodeling, metabolic reprogramming, and malignant progression. The identification of *MAP1B* as a functional driver further underscores the translational potential of our findings. Together, these results enrich the understanding of GBM pathogenesis and may guide the development of novel targeted and immunotherapeutic strategies.

## Data Availability

The original contributions presented in the study are included in the article/**Supplementary Material**. Further inquiries can be directed to the corresponding author.

## Supplementary material

Supplementary Figure 1 Pyroptosis activity scoring at the single-cell level in GBM. **(A)** Bubble plot showing the distribution of pyroptosis scores across major cell types, calculated using five independent methods: AUCell, UCell, singscore, ssGSEA, and AddModuleScore. **(B)** UMAP projection illustrating the overall average pyroptosis scores of all cells. **(C)** Violin plots displaying the differences in pyroptosis activity among distinct cell populations.

Supplementary Figure 2 Slingshot pseudotime analysis and transcription factor regulatory network. **(A)** Two differentiation trajectories identified by Slingshot, illustrating the dynamic distribution of malignant epithelial cells along pseudotime. **(B)** Slingshot trajectories overlaid on the UMAP space, showing the origin and differentiation directions across four malignant clusters. **(C)** Functional enrichment analysis based on the SCP package, highlighting representative biological pathways associated with key gene sets along the two trajectories. **(D)** Heatmap of transcription factor activities inferred by SCENIC, demonstrating differential sensitivity of malignant epithelial subpopulations to core regulatory factors.

Supplementary Figure 3 Identification of prognostic genes and batch effect correction. **(A)** Differentially expressed genes between malignant epithelial cells with distinct pyroptosis activity were identified, followed by univariate Cox regression to screen candidate genes significantly associated with prognosis in GBM. Principal component analysis (PCA) plots of samples from CGGA, TCGA, GSE13041, GSE74187, and GSE83300 cohorts before **(B)** and after **(C)** batch effect correction using the sva package. The improved overlap after correction indicates effective removal of batch effects across datasets.

Supplementary Figure 4 Expression patterns of PRGS genes at the single-cell level.In the integrated GBM single-cell dataset, expression of the PRGS component genes (*PDLIM4, MAP1B, MGP, POSTN, LGALS1, CCL2, ANXA1, MYL12A, CLIC1, and GSN*) is shown. Each panel displays a UMAP visualization, where color intensity represents expression levels, confirming the distribution of signature genes across distinct cellular populations.
