# Supplementary figures and images for: Single-cell and machine learning-based pyroptosis-related gene signature predicts prognosis and immunotherapy response in glioblastoma
Source: Front Immunol. 2025 Oct 24;16:1693940. doi: 10.3389/fimmu.2025.1693940 (PMC12592165; doi:10.3389/fimmu.2025.1693940)

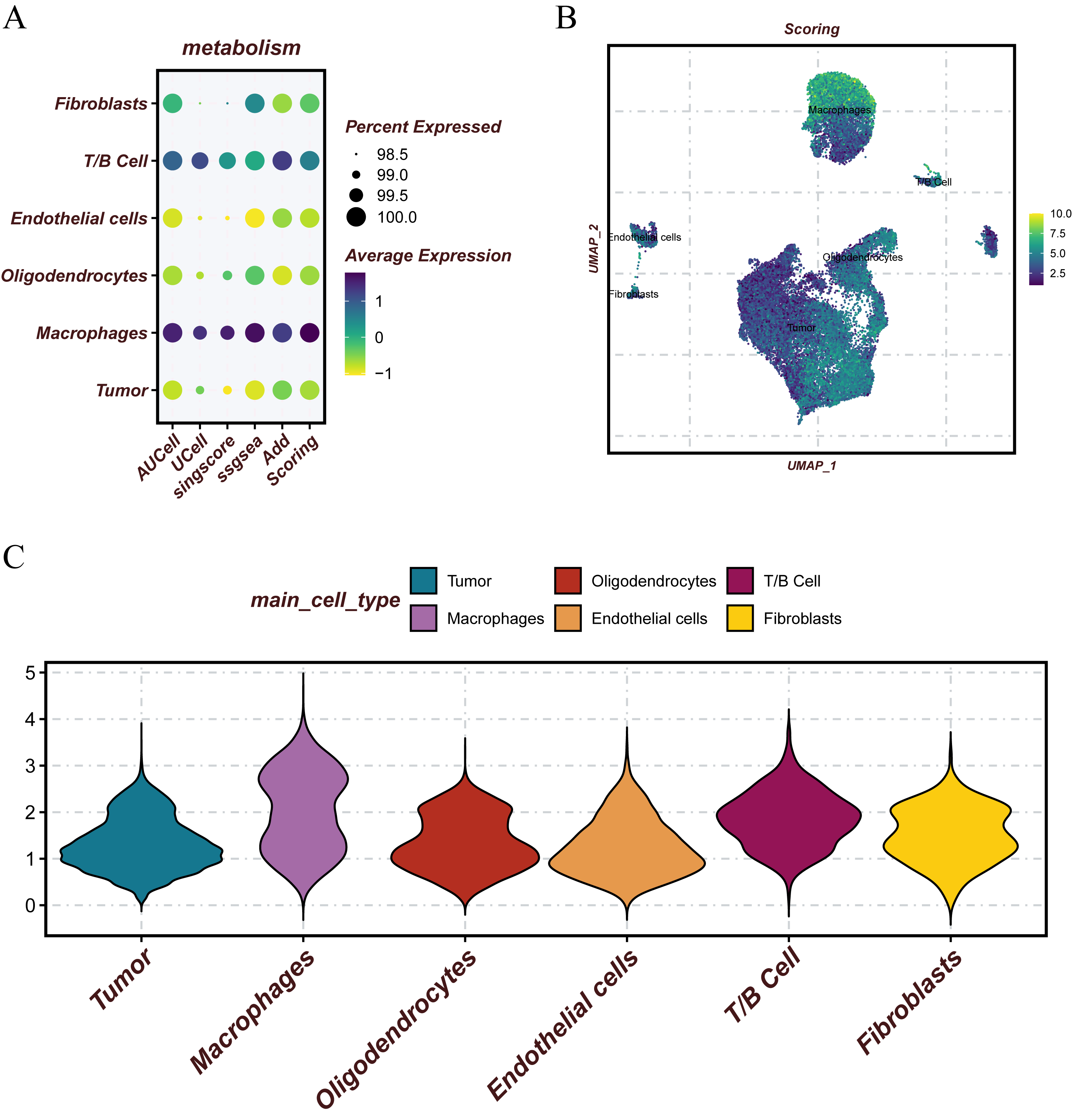

Supplement: Supplementary Figure 1 — Pyroptosis activity scoring at the single-cell level in GBM. (A) Bubble plot showing the distribution of pyroptosis scores across major cell types, calculated using five independent methods: AUCell, UCell, singscore, ssGSEA, and AddModuleScore. (B) UMAP projection illustrating the overall average pyroptosis scores of all cells. (C) Violin plots displaying the differences in pyroptosis activity among distinct cell populations. [file Image1.tif]

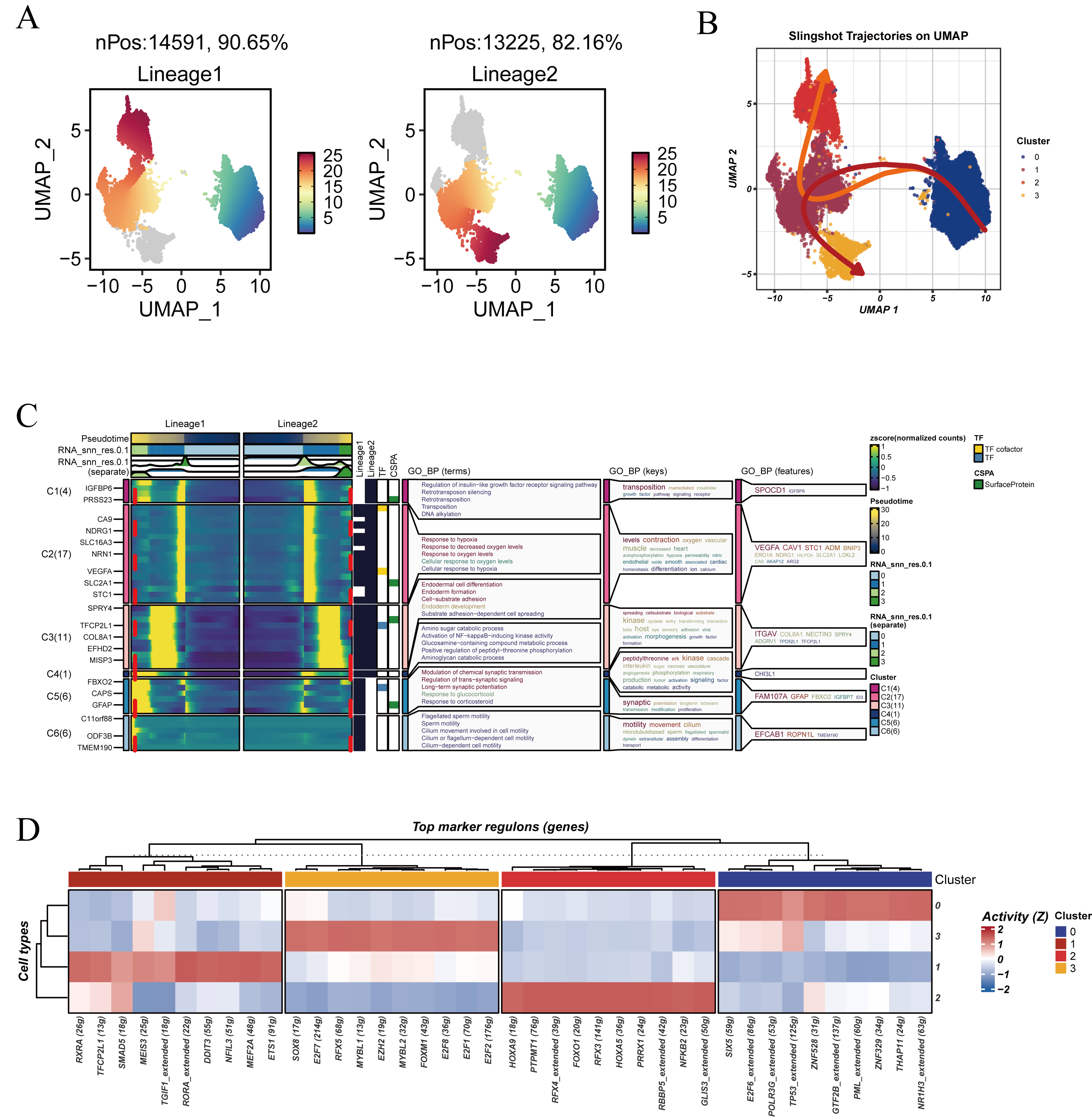

Supplement: Supplementary Figure 2 — Slingshot pseudotime analysis and transcription factor regulatory network. (A) Two differentiation trajectories identified by Slingshot, illustrating the dynamic distribution of malignant epithelial cells along pseudotime. (B) Slingshot trajectories overlaid on the UMAP space, showing the origin and differentiation directions across four malignant clusters. (C) Functional enrichment analysis based on the SCP package, highlighting representative biological pathways associated with key gene sets along the two trajectories. (D) Heatmap of transcription factor activities inferred by SCENIC, demonstrating differential sensitivity of malignant epithelial subpopulations to core regulatory factors. [file Image2.tif]

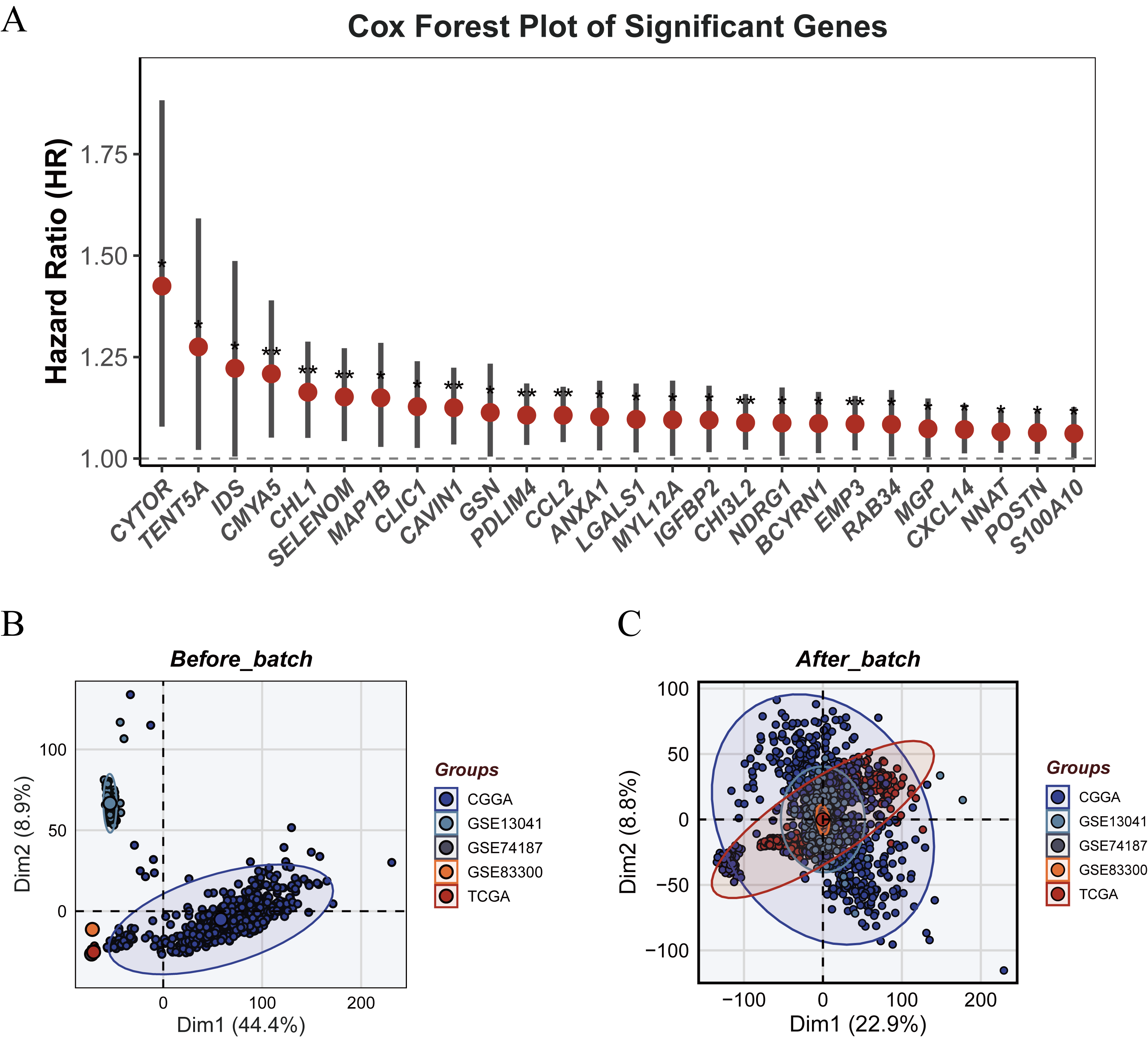

Supplement: Supplementary Figure 3 — Identification of prognostic genes and batch effect correction. (A) Differentially expressed genes between malignant epithelial cells with distinct pyroptosis activity were identified, followed by univariate Cox regression to screen candidate genes significantly associated with prognosis in GBM. Principal component analysis (PCA) plots of samples from CGGA, TCGA, GSE13041, GSE74187, and GSE83300 cohorts before (B) and after (C) batch effect correction using the sva package. The improved overlap after correction indicates effective removal of batch effects across datasets. [file Image3.tif]

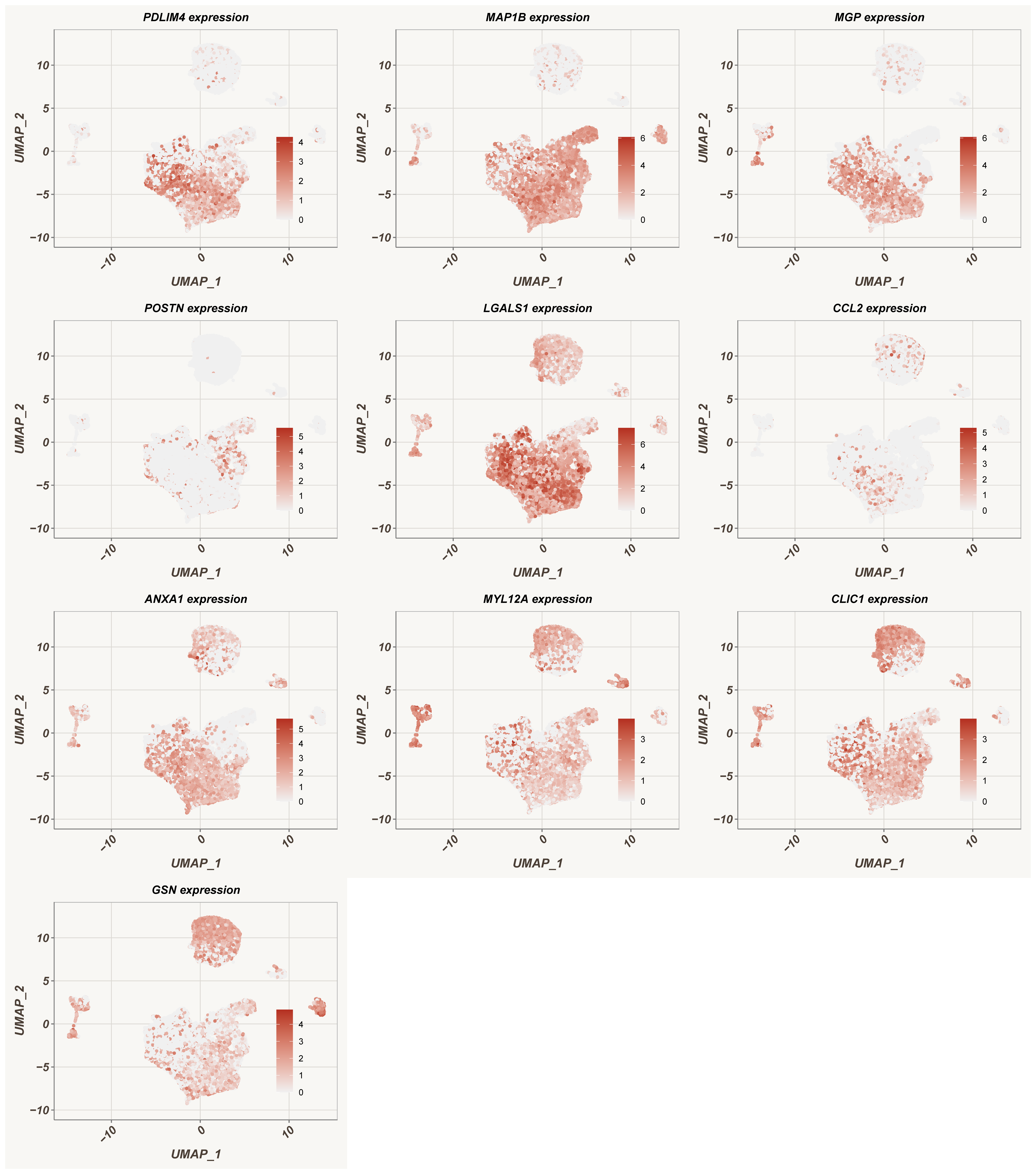

Supplement: Supplementary Figure 4 — Expression patterns of PRGS genes at the single-cell level.In the integrated GBM single-cell dataset, expression of the PRGS component genes (PDLIM4, MAP1B, MGP, POSTN, LGALS1, CCL2, ANXA1, MYL12A, CLIC1, and GSN) is shown. Each panel displays a UMAP visualization, where color intensity represents expression levels, confirming the distribution of signature genes across distinct cellular populations. [file Image4.tif]

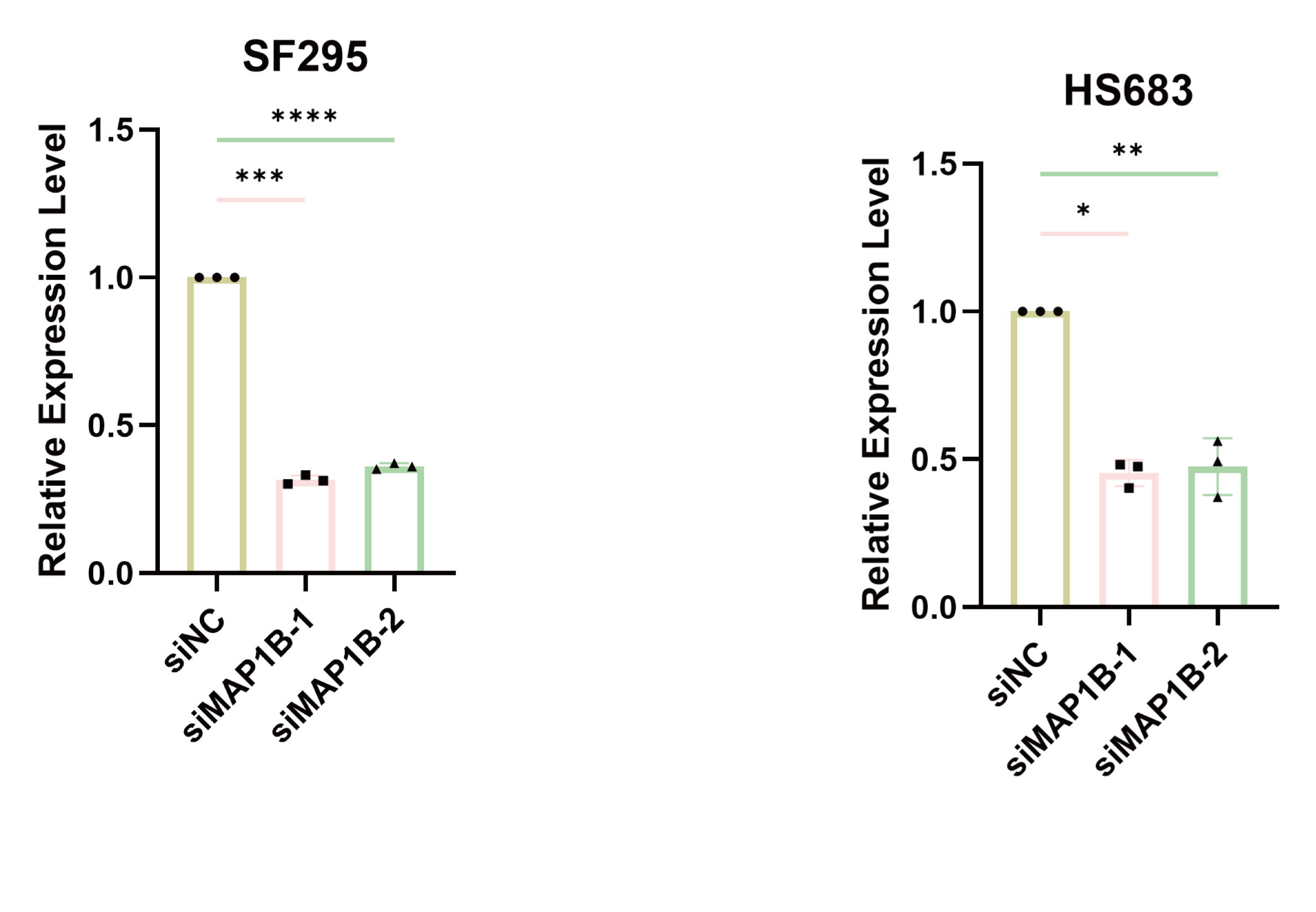

Supplement: Supplementary file 5 [file Image5.tif]
